# Structural basis of the fanconi anemia-associated mutations within the FANCA and FANCG complex

**DOI:** 10.1093/nar/gkaa062

**Published:** 2020-01-31

**Authors:** Eunyoung Jeong, Seong-Gyu Lee, Hyun-Suk Kim, Jihyeon Yang, Jinwoo Shin, Youngran Kim, Jihan Kim, Orlando D Schärer, Youngjin Kim, Jung-Eun Yeo, Ho Min Kim, Yunje Cho

**Affiliations:** 1 Department of Life Science, Pohang University of Science and Technology, Pohang, 37673, Republic of Korea; 2 Center for Biomolecular and Cellular Structure, Institute for Basic Science (IBS), Daejeon, 34141, Republic of Korea; 3 Graduate School of Medical Science & Engineering, Korea Advanced Institute of Science and Technology, Daejeon, 34141, Republic of Korea; 4 Center for Genomic Integrity, Institute for Basic Science (IBS), Ulsan, 44919, Republic of Korea; 5 Department of Biological Sciences, School of Life Sciences, Ulsan National Institute of Science and Technology (UNIST), Ulsan, 44919, Republic of Korea

## Abstract

Monoubiquitination of the Fanconi anemia complementation group D2 (FANCD2) protein by the FA core ubiquitin ligase complex is the central event in the FA pathway. FANCA and FANCG play major roles in the nuclear localization of the FA core complex. Mutations of these two genes are the most frequently observed genetic alterations in FA patients, and most point mutations in FANCA are clustered in the C-terminal domain (CTD). To understand the basis of the FA-associated FANCA mutations, we determined the cryo-electron microscopy (EM) structures of *Xenopus laevis* FANCA alone at 3.35 Å and 3.46 Å resolution and two distinct FANCA–FANCG complexes at 4.59 and 4.84 Å resolution, respectively. The FANCA CTD adopts an arc-shaped solenoid structure that forms a pseudo-symmetric dimer through its outer surface. FA- and cancer-associated point mutations are widely distributed over the CTD. The two different complex structures capture independent interactions of FANCG with either FANCA C-terminal HEAT repeats, or the N-terminal region. We show that mutations that disturb either of these two interactions prevent the nuclear localization of FANCA, thereby leading to an FA pathway defect. The structure provides insights into the function of FANCA CTD, and provides a framework for understanding FA- and cancer-associated mutations.

## INTRODUCTION

Fanconi anemia (FA) is a genetic disorder characterized by cellular hypersensitivity toward DNA interstrand crosslinking (ICL) reagents, bone marrow failure, developmental defects and cancer susceptibility (1–3). The FA pathway is triggered by stalled DNA replication forks at ICLs, leading to the activation of the FA core ubiquitin ligase complex (4,5). The FA complementation (FANC) core complex is imported into the nucleus and catalyzes the addition of a single ubiquitin to each chain of the FANCI–FANCD2 (ID) complex. Ubiquitinated ID localizes to chromatin, leading to the recruitment of nucleases that unhook the ICLs in preparation for repair by translesion synthesis and homologous recombination (6–12).

The FA core complex comprises seven FANC proteins and two FA-associated proteins (FAAPs) that form a network of three functional modules (6,13–14). A dimer of the FANCB-FANCL-FAAP100 (BL100) hetero-trimer at the center of the core complex provides the catalytic ubiquitin ligase activity and forms a scaffold for other subcomplexes (13–15). The FANCC-FANCE-FANCF (CEF) complex anchors on the BL100 complex and transfers the ID substrate to the FANCL ligase (16–20). The BL100 and CEF subcomplexes are sufficient to monoubiquitinate FANCD2 *in vitro* (13,14). However, the FANCA–FANCG–FAAP20 (AG20) subcomplex is essential for monoubiquitination of FANCD2 *in vivo* (6).

**Figure 1.**
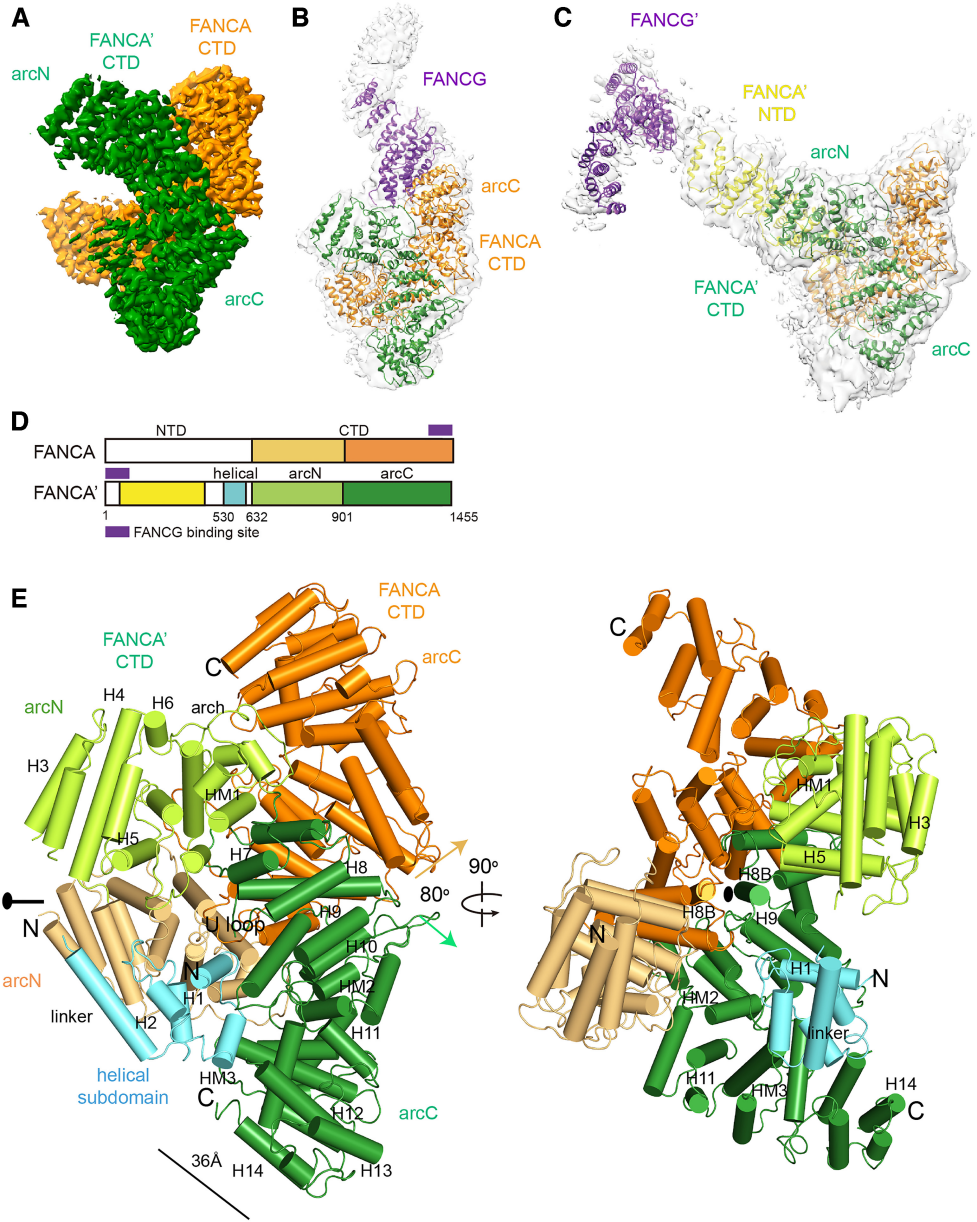
Three classes of the overall structures of the FANCA–FANCG complex. (**A**) Cryo-EM map of the FANCA CTD dimer at an average resolution of 3.35 Å. Each FANCA molecule is shown in orange (FANCA) and green (FANCA’). The arc-shaped CTD is divided into the N-terminal half (arc^N^) and the C-terminal half (arc^C^). (**B**) Cryo-EM map (gray) of the FANCA CTD dimer complexed with a FANCG (purple) molecule at 4.6 Å. FANCG CTD packs at the C-terminal end of FANCA. Orientation of the CTD dimer is same as that in (A). (**C**) Cryo-EM map (gray) of the FANCA and FANCG complex at 4.84 Å. FANCG’ is bound at the FANCA’ NTD (yellow). (**D**) Overall scheme of the subdomain composition in FANCA and FANCA’. Each subdomain is painted with same colors as in (A) to (E). (**E**) Two views of the 3.46 Å structure of the FANCA CTD dimer. Left, A 2-fold rotation axis is along the horizontal axis. Each HEAT repeat consists of the HA and HB helices that forms inner and outer surfaces, respectively. The central axes (along each H8B helix) of two solenoids are arranged in ∼80°. Right, View orthogonal to the left view looking down the 2-fold axis which is along the H8B helices from the two FANCA molecules. The CTD structure is divided into the N-terminal helical subdomain (H1 to linker helix), arc^N^ (H3 to HM1) and arc^C^ (H7 to H14) subdomains. The helical subdomain of FANCA’ is shown in cyan. The arc^N^ and arc^C^ subdomains of FANCA CTD are colored in light orange and orange, respectively. The arc^N^ and arc^C^ subdomains of FANCA’ CTD are shown in light green and green, respectively.

FANCA and FANCG are critical for the localization of the FANC core complex (21,22). FANCA contains a bipartite nuclear localization signal (NLS) motif in the N-terminal region, which is recognized by FANCG, a critical feature for nuclear accumulation of the FANC core proteins (23,24). Other FANC core components are also required for nuclear import of FANCA, suggesting that the three functional modules of the FANC core cooperate to achieve nuclear localization (25–27). Interestingly, FANCA mutants lacking the C-terminal domain (CTD) were able to form a complex with FANCG, but failed to be imported into the nucleus, suggesting that the CTD of FANCA is crucial for localization of the FANC core complex (24). Most FA- and cancer-associated point mutations in FANCA are clustered at the CTD, supporting the importance of this structural component for FANCA function (http://www2.rockefeller.edu/fanconi/ and https://cancer.sanger.ac.uk/cosmic/gene/analysis?ln=FANCA). The CTD binds to single-strand DNA (ssDNA) and ssRNA, and it has been suggested that the CTD contributes to the ssDNA annealing (SSA) and exchange (SSE) activities of FANCA (28,29). Dimerization of FANCA is important for these activities (28). To investigate the roles of FANCA and FANCG in the FA pathway, and to better understand the FA- and cancer-associated mutations, we determined several cryo-electron microscopy (EM) structures of *Xenopus laevis* FANCA: the FANCA CTD alone at 3.35 Å and 3.46 Å resolution and the two distinct FANCA–FANCG complexes at 4.59 and 4.84 Å resolution, respectively (Figure 1; Supplementary Figures S1-2 and Table S1).

The two structures of FANCA alone show that the FANCA CTD folds into an arc-shaped solenoid structure, which forms a pseudo-symmetric dimer. The structure allows for the establishment of a correlation in the patient-derived mutations, the nuclear localization, stability and the DNA binding in FANCA. The FANCA CTD structure reveals a long path decorated with a cluster of positively charged residues, providing a rationale for how FANCA might interact with DNA. The structure of the FANCA complexed with FANCG (FANCA–FANCG) reveals that the C-terminal HEAT (**H**untingtin, elongation factor 3 (**E**F3), protein phosphatase 2A (PP2**A**), and the yeast kinase **T**OR1) repeats of FANCA binds to the helical repeats at the C-terminal end of FANCG (Figure 1B and D). In another complex structure (FANCA’–FANCG’), the N-terminal domain (NTD) of the full-length FANCA interacts with FANCG (Figure 1C and D). Both binding interfaces are required for the nuclear localization of FANCA. We present a model of the FANCA and FANCG complex as a hetero-tetramer, in which the FANCG–FANCA–FANCA’–FANCG’ forms a curved chain. Our structures provide insights into the function of the FANCA CTD and a framework for understanding how the FA- and cancer-associated missense mutations affect the activity of the FA pathway.

## MATERIALS AND METHODS

### Expression and purification of the FANCA–FANCG complex

Genes encoding *Xenopus laevis* (*Xl*) FANCA and FANCG were synthesized with codon-optimization for expression in insect cells (Gene Universal). Genes encoding full-length FANCA and FANCG were amplified by polymerase chain reaction (PCR) and separately cloned pFastBac vectors (EcoRI–NotI for FANCA and EcoRI–HindIII for FANCG). The resulting FANCA and FANCG constructs were fused with a Strep tag (WSHPQFEK) and a 2 × Flag tag, respectively, at their N-terminal end. Baculoviruses harboring FANCA and FANCG constructs were generated in *Spodoptera frugiperda* Sf9 cells using the Bac-to-Bac system (Invitrogen). For expression of the FANCA–FANCG complex, High-Five cells were co-infected with both baculoviruses at a multiplicity of infection (MOI) of 3.5 and harvested after 60 h. Cell pellets were resuspended in lysis buffer (25 mM Tris–HCl pH 8.0, 0.5 M NaCl, 5 mM dithiothreitol (DTT), 10% glycerol, 1 mM phenylmethylsulfonyl fluoride (PMSF), Roche protease inhibitor cocktail) and sonicated. The lysate was cleared by ultracentrifugation at 40 000 *g* and the supernatant was applied to StrepTactin XT resin (IBA Lifesciences). After washing, the bound protein was eluted with Strep elution buffer (25 mM Tris–HCl pH 8.0, 0.5 M NaCl, 5 mM DTT, 10% glycerol and 50 mM biotin) and subsequently loaded onto a Mono Q 10/100 GL column for ion-exchange chromatography (GE Healthcare). The column was washed carefully and eluted over a 0−600 mM NaCl linear gradient. Peak fractions were collected, concentrated, and stored at −80°C.

### Cryo-EM sample preparation and data collection

The FANCA–FANCG complex was freshly prepared for cryo-EM grids immediately after size exclusion chromatography, which was performed using a Superdex 200 10/300 GL column equilibrated with 25 mM Tris–HCl pH 8.0, 0.2 M NaCl and 2 mM DTT (GE Healthcare). Peak fractions were collected, mixed with 0.003% n-dodecyl-β-D-maltopyranoside (DDM) and 3 μl of the complex (0.8 mg/ml) was applied to a glow-discharged holey carbon grid (C-flat 1.2/1.3 Au 400-mesh grid; EMS). Grids were plunge-frozen in liquid ethane using a Vitrobot Mark IV (Thermo Fisher Scientific) operating at 4°C with 100% humidity and blotted for 8.5 s with the blot force 2 setting. Images were acquired using an FEI Titan Krios transmission electron microscope operated at 300 kV and equipped with an FEI Falcon III direct electron detector (at Korea Basic Science Institute) at a nominal magnification of 47 000 ×, corresponding to a pixel size of 1.4 Å at the specimen level.

Two datasets were collected comprising 2176 (dataset I) and 1903 (dataset II) micrographs. Both datasets were collected with a pixel size of 1.4 Å and a total electron dose of 30 e^−^/Å^2^. A defocus of −1.5 to −2.5 μm was employed, 30 frames were collected in 74.4 s (2.48 s/frame) and the total electron dose was 30 electrons per Å^2^ distributed over 30 frames (Supplementary Table S1).

### Data processing

Movie frames were aligned and dose-weighted using MotionCor2 (30). The resulting motion-corrected sums were used to estimate the contrast transfer function (CTF) using CTFFIND4 (31). Micrographs with low estimated resolution were eliminated. Initially, ∼4000 particles were manually picked using cryoSPARC v.2 to generate two-dimensional (2D) models for reference-based particle picking (32). Manually picked particles were subjected to several rounds of unsupervised 2D classification. A total of 1 523 728 particles were automatically picked from 3635 micrographs based on the generated reference models and extracted with a box size of 256 pixels (Supplementary Figure S1). Contaminants and false-positive particles were removed by rounds of 2D classification, and 501 654 particles were retained for further processing. This particle set was subjected to *ab initio* reconstruction and 3D heterogeneous refinement into four classes with C1 symmetry. Both two classes revealed a clear arc-shaped 3D volume and most particles were selected for 3D homogeneous refinement. The arc-shaped 3D volume contains residues 631–1402 of FANCA (see below). The combined classes yielded 306,460 particles and were further improved by performing local motion correction and patch CTF extraction. Subsequent homogeneous refinement and non-uniform refinement of polished particles resulted in a map with a global resolution of 3.35 Å according to a Fourier shell correlation (FSC) criterion of 0.143. One of the selected classes showing additional small density corresponding to helical subdomain (residues 530–619) of FANCA was subjected to homogeneous refinement followed by non-uniform refinement which resulted in a resolution of 3.46 Å after particle polishing. The final map was sharpened using negative B-factors automatically determined by cryoSPARC v.2. The other two classes, one with elongated density between the two arc^N^ subdomains (Because FANCA CTD forms an arc-shaped structure, we refer the N-terminal half (residues 631–900) and the C-terminal half (residues 901–1402) of FANCA CTD to arc^N^ and arc^C^, respectively, throughout the text) and the other with superhelical density beneath the arc-shaped volume, were exported to RELION-3 (33) and subjected to 3D refinement. The resulting two classes with different shaped molecules were further classified by focussed 3D classification with a mask covering additional density not including the arc-shaped volume. Each of the two classes of particles were divided into five and six subsets, respectively. In each class, one subset with different conformation from the others was excluded. The remaining subsets were combined for 3D refinement and post-processing, which resulted the two complex structures in resolutions of 4.59 Å and 4.84 Å, respectively, corresponding to an FSC value of 0.143 after particle polishing (Supplementary Figure S1). The maps were sharpened using negative B-factors automatically determined by RELION-3.

### Model building

An atomic model was built from the 3.35 Å EM map, revealing a helix-rich molecule with a solenoid shape. The map was of good quality and clearly revealed bulky side chains and good connectivity for the main chains, which allowed us to assign most of the side chains. Overall, the C-terminal half of FANCA clearly fits into the EM map. Initially, FANCA was built *de novo* from a poly-Ala model, which was manually improved using COOT (34). Sequence assignment was guided by bulky Tyr, Phe and Trp residues, and small Gly, Ser and Val residues. There was no clear density for the N-terminal 20 residues or the C-terminal 20 residues, hence we could not assign side chains for these residues. The structure was subjected to real-space refinement using PHENIX 1.14 with secondary structures and geometry restraints (35). Iteration of model building and refinement yielded the final model that includes residues 631–1402. The refined model has a MolProbity (36) score of 1.84 and a Clash score of 4.17 at 3.8 Å resolution with excellent geometry (Supplementary Table S1). A model for the helical subdomain (residues 530–619) of one FANCA molecule was built on the 3.46 Å EM map. The regions modeled as poly-alanine chains or omitted are summarized in Supplementary Table S3

In other models, the NTD of FANCA (FANCA’) and FANCG bound to FANCA CTD (FANCA) were built as poly-alanine chains in COOT. The NTD of FANCA and FANCG bound to FANCA CTD were assigned with 310 and 319 alanine residues, respectively. Because the EM map of FANCG bound to the FANCA NTD was not well-resolved, we docked a FANCG model from the Passmore's group (PDB ID: 6SRI) into the EM map in Chimera using ‘fit in map’ tool (15,37). However, the EM map of FANCG CTD bound to FANCA CTD was more clearly resolved and we were able to independently build a model in *ab initio*.

### FANCA cross-linking assay

The purified FANCA and FANCG hetero-tetramer was diluted to 0.2 mg/ml with reaction buffer (25 mM Tris–HCl pH 7.0, 0.2 M NaCl and 5% glycerol), resulting a final concentration of 0.38 mM DTT. Bismaleimidoethane (BMOE; Thermo Fisher Scientific) was dissolved in dimethylsulfoxide (DMSO) immediately before use and added at the indicated concentrations. After a 5 min incubation at room temperature, the reaction was quenched by addition of 70 mM cysteine and further incubated for 10 min. Samples from stopped reactions were subjected to sodium dodecyl sulfate polyacrylamide gel electrophoresis (SDS-PAGE). For H_2_O_2_ crosslinking reactions, DTT in the purified FANCA and FANCG complex was completely removed by dialysis against 25 mM Tris–HCl pH 7.0, 0.2 M NaCl and 10% glycerol. The dialyzed sample was incubated with H_2_O_2_ at room temperature. After a 3 h incubation, samples were directly separated by SDS-PAGE then subjected to Coomassie Brilliant Blue staining.

### Yeast two-hybrid assay

Yeast two-hybrid assays were performed by Panbionet (http://panbionet.com). The synthesized genes encoding human FANCA and FANCG were amplified by PCR and cloned into both pGBKT7 and pGADT7 vectors (Clontech). All the FANCA mutations used in this study were introduced by PCR-based site-directed mutagenesis and verified by DNA sequencing. Plasmids were transformed into the AH109 yeast strain, which expresses HIS3 reporters. Transformants were separately plated on SD-LeuTrp and SD- LeuTrpHis medium containing 10 mM 3-amino-1,2,3-triazole (3-AT), a competitive inhibitor of the HIS3 protein (His3p).

### Immunofluorescence microscopy

The gene encoding human FANCA was amplified and cloned into the pFlag-CMV-2 vector (a generous gift from Dr Kyong Tai Kim at POSTECH) using EcoRI and XbaI. The full length FANCA construct was fused with a Flag tag at its N-terminal end. The mutations were introduced in the same way mentioned above. The FANCA-deficient U2OS cell line was a generous gift from Dr Yanbin Zhang at University of Miami (28,38). Transfections for Transient expression of FANCA WT and mutant constructs were performed using Lipofectamine 3000 transfection reagent according to the manufacturer's protocol. Transfected cells were treated with either H_2_O (Mock) or 1uM mitomycin C (MMC) for 24 h and then washed with phosphate-buffered saline (PBS, pH 7.4). Cells were fixed with 4% paraformaldehyde in PBS, followed by washing four times with 0.1% triton X-100 in PBS for 5 min each on a shaker. Next, the cells were incubated for 1 h in blocking buffer (3% BSA in PBS), followed by incubation in blocking buffer containing anti-FANCA antibody (1:250, Rabbit, Bethyl, A301–980A) or anti-Flag antibody (Cell Signaling, 8146S) for 2 h at room temperature. Cells were washed four times with 0.1% triton X-100 in PBS for 5 min each on a shaker and incubated for 1 h in blocking buffer containing goat anti-mouse secondary antibody conjugated to Alex488 (Invitrogen). Cells were again washed with washing buffer. Cell nuclei were stained with a mounting medium containing 4′,6-diamidino-2-pheylindole (DAPI) (Vector Laboratories, CA, USA). For analysis of FANCA localization, images were taken using a ZEISS Axio observer seven microscopy. To determine percentages of cells with different staining patterns, 100 cells were scored in two independent experiments.

## RESULTS

### Structures of the FANCA and FANCG complex

Size exclusion chromatography analysis showed that FANCA and FANCG form a stable complex with a molecular weight of ∼500 kDa that is predicted to be a hetero-tetramer (Supplementary Figure S1). Single-particle cryo-EM analysis revealed four classes of FANCA–FANCG complex structures—two classes for FANCA alone and two classes for the complex. One of the major classes corresponds to the dimer of the CTD of FANCA (Figure 1A and E; Supplementary Figures S1 and 2). The side chains of FANCA are well-defined in the 3.35 Å EM density map, and clusters of bulky hydrophobic side chains unambiguously guided the tracing of residues 631–1370 (helix H3A to helix H13B, Supplementary Figures S2-3 and Table S3). The model is supported by *in vitro* analyses showing that FANCA dimerizes through its CTD, and FANCA CTD can be cross-linked through the proximal Cys residues at the dimeric interface (28,39) (Supplementary Figure S4). Two additional helices are also clearly visible at the C-terminal ends (H14A and H14B) of FANCA CTD. However, the side chains of the residues for these helices are not clearly defined. The N-terminal helical subdomain (residues 530–619) is visible only in one FANCA CTD protomer (FANCA’) in the 3.46 Å EM density map (Supplementary Figure S1 and Table S3). FANCA recognizes FANCG through two different modes. In one structural class of the complex, one of the protomers (orange color) in a FANCA dimer packs against the tetratricopeptide repeat (TPR) region of FANCG (purple) through its C-terminal HEAT repeats (Figure 1B and Supplementary Figure S1). We refer this complex as the FANCA–FANCG complex throughout the text. In the second class of the FANCA–FANCG complex, the NTD of FANCA (yellow) formed with multiple HEAT repeats is packed at the inner surface of the FANCA dimer, through which FANCG (purple) is recruited (Figure 1C and Supplementary Figure S1). We define this complex as the FANCA’–FANCG’ complex.

### Overall structure of FANCA

The FANCA CTD folds into an arc-shaped α-solenoid structure (light orange and orange color) (Figure 1A, D and E). The radius of the arc is 36 Å, and the longest dimension is 115 Å. The FANCA CTD structure consists of a chimera of 14 HEAT repeats and three helical motifs (HMs) with two to four helices. The FANCA CTD can be divided into three subdomains (Figure 1D and E; Supplementary Figure S3); the N-terminal helical subdomain inside the arc (residues 530–619, cyan), the N-terminal half (arc^N^, residues 632–900, FANCA in light orange and FANCA’ in light green) and C-terminal half (arc^C^, 901–1403, FANCA in orange and FANCA’ in green) of the arc-shaped CTD (Figure 1D and E). The helical subdomain which is visible only in FANCA’ is packed against the arc^C^ subdomain and is connected to the HEAT 3 repeat through the linker helix. The loop between the linker helix and the H3A helix is disordered, suggesting that this region can undergo dynamic movement. Although the two FANCA CTD molecules share similar structures, one of them (FANCA CTD, orange) is slightly more open than the other (FANCA’ CTD, green), with a root mean square deviation (rmsd) value of 2.2 Å when all Cα atoms of the arc domains are aligned (Supplementary Figure S5). The loops connecting the helices within a repeat (intra-repeat loops) as well as adjacent repeats (inter-repeat loops) vary in length and conformation, resulting in the formation of surfaces where another CTD or NTD of FANCA or FANCG binds.

### Pseudo-symmetric dimeric interface between the two FANCA CTD molecules

**Figure 2.**
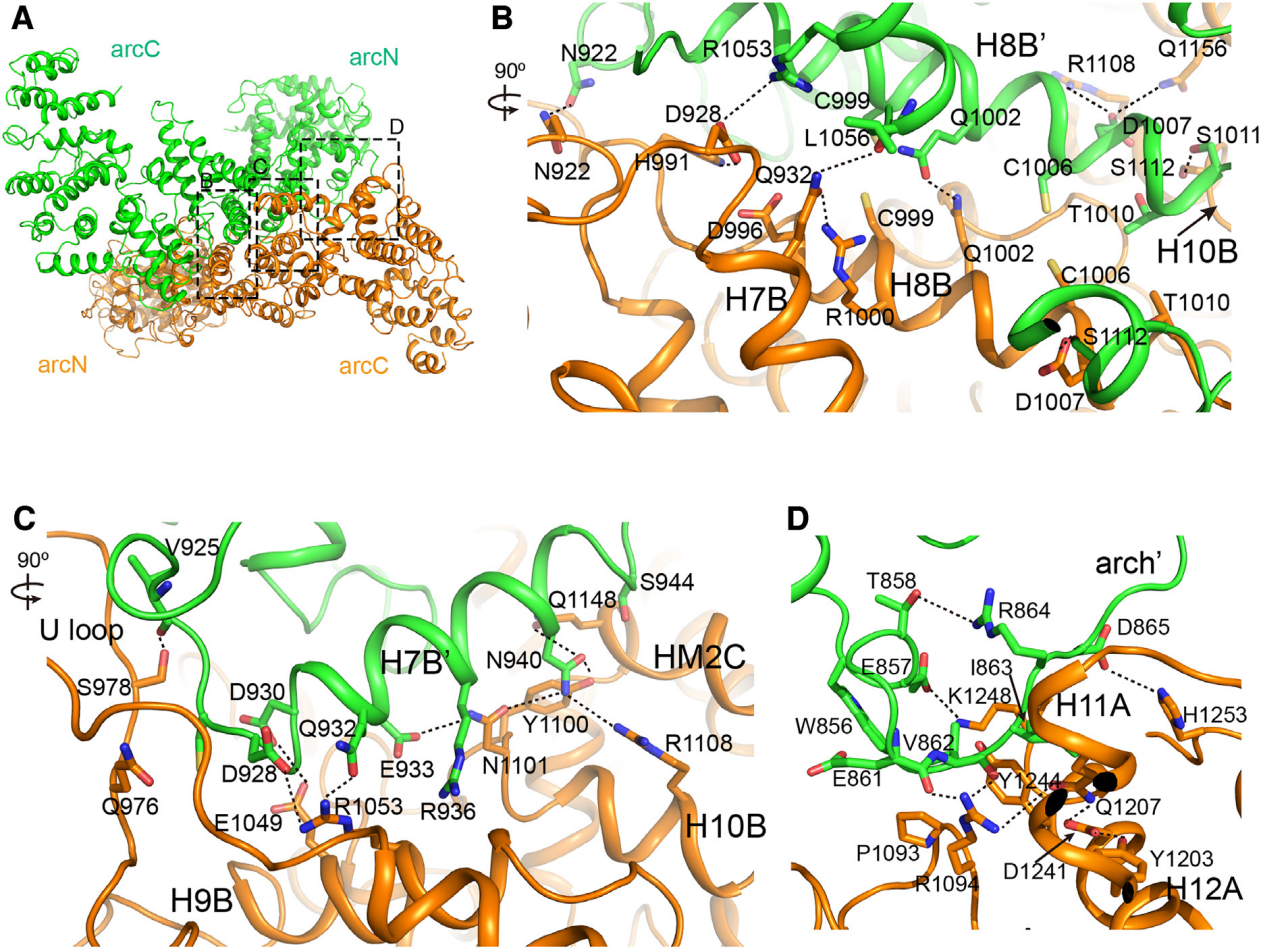
The dimer interface of FANCA. (**A**) View looking down the dimer interface between the arc^C^ of FANCA CTD (orange) and the arc^N^ of FANCA’ CTD (green). Boxes contain clusters of contacts with their respective segments labeled. (**B**) Close-up view of the central interface in box b from (A). A view is orthogonal to the projection in (A). Black dotted lines indicate polar interactions. The interface is formed by the interactions between the H8B and the H8B’ helices and between the H8B’ and the H10B helices. (**C**) Close-up view of the interface between the H7B’ helix from FANCA’ CTD and the H9B, H10B and HM2C from FANCA CTD. A view is orthogonal to the projection in (A). The interface is formed primarily by the hydrogen bonds and polar interactions between the main chain and side chain. (**D**) Close-up view of the interface between the arch’ loop and the H11A and H12A helices in the same projection of (A).

The convex surface forms a wide and shallow groove (44 Å width, 35 Å length, 6 Å depth), through which the extensive dimer interface (6238 Å^2^) is created. The two FANCA CTDs interact in a head-to-tail manner, in which the arc^C^ subdomain of FANCA’ CTD (green) interacts with the arc^N^ subdomain of FANCA CTD (orange; Figures 1E and 2A). The dimeric interface between the two FANCA CTD molecules includes an extended network of interactions involving more than 40 H-bonds, 10 ion-pairs and extensive hydrophobic and van der Waals contacts.

At the center of the dimer, two parallel H8B helices run along the 2-fold rotation axis (Figure 2A and B). Two Cys pairs (C999-C999 and C1006-C1006) are in close proximity, consistent with the results of the intermolecular crosslinking analysis using hydrogen peroxide and bis-maleimidoethane (Figure 2B and Supplementary Figure S4). Two H-bonds (Gln1002-Gln1002 and Asn922-Asn922) and one ion pair (Asp928-Arg1053) further stabilize this interface, which extends continuously in both directions. On the right side of the center, H7B’ from FANCA’ CTD forms another interface with three helices (H9B, H10B and HM2C) from FANCA CTD (Figure 2A and C). Polar interactions (eight H-bonds and an ion pair) dominate this interface. A third interface is formed between the arch’ loop of FANCA’ CTD and H11A and H12A near the end of FANCA CTD (Figure 2D). However, these interactions are not formed at the reciprocal end, exhibiting the pseudo-symmetrical nature of a CTD dimer.

Many residues at the interface between the CTD dimer are mutated in FA- patients and cancer cells (Supplementary Figure S3), pointing to the significance of the dimerization. A double mutation of Leu1069 and Leu1076 (Leu1042 and Leu1048 in *Xl* FANCA) to Ala disrupted dimerization and abrogated the SSA activity of FANCA (28). Leu1048 is packed against Leu1029, Leu1049 and Cys1051 near the dimeric interface (Supplementary Figure S6A). Arg951Trp (Arg931 in *Xl* FANCA) and Gln1128Glu (Tyr1100) mutations decreased SSA activity by 40% (28). These two residues are near the dimeric interface. Arg931 forms an ion pair with Asp996, which is near Ser990 of another CTD molecule (Supplementary Figure S6B), while Tyr1100 is packed against Gln1148 and Leu1149, and within 4 Å from Asn940 of an adjacent CTD molecule (Figure 2C and Supplementary Figure S6C). Thus, many residues mutated in FA patients are at the dimeric interface and tightly correlated with SSA and SSE activities, confirming the importance of FANCA dimerization for these activities.

**Figure 3.**
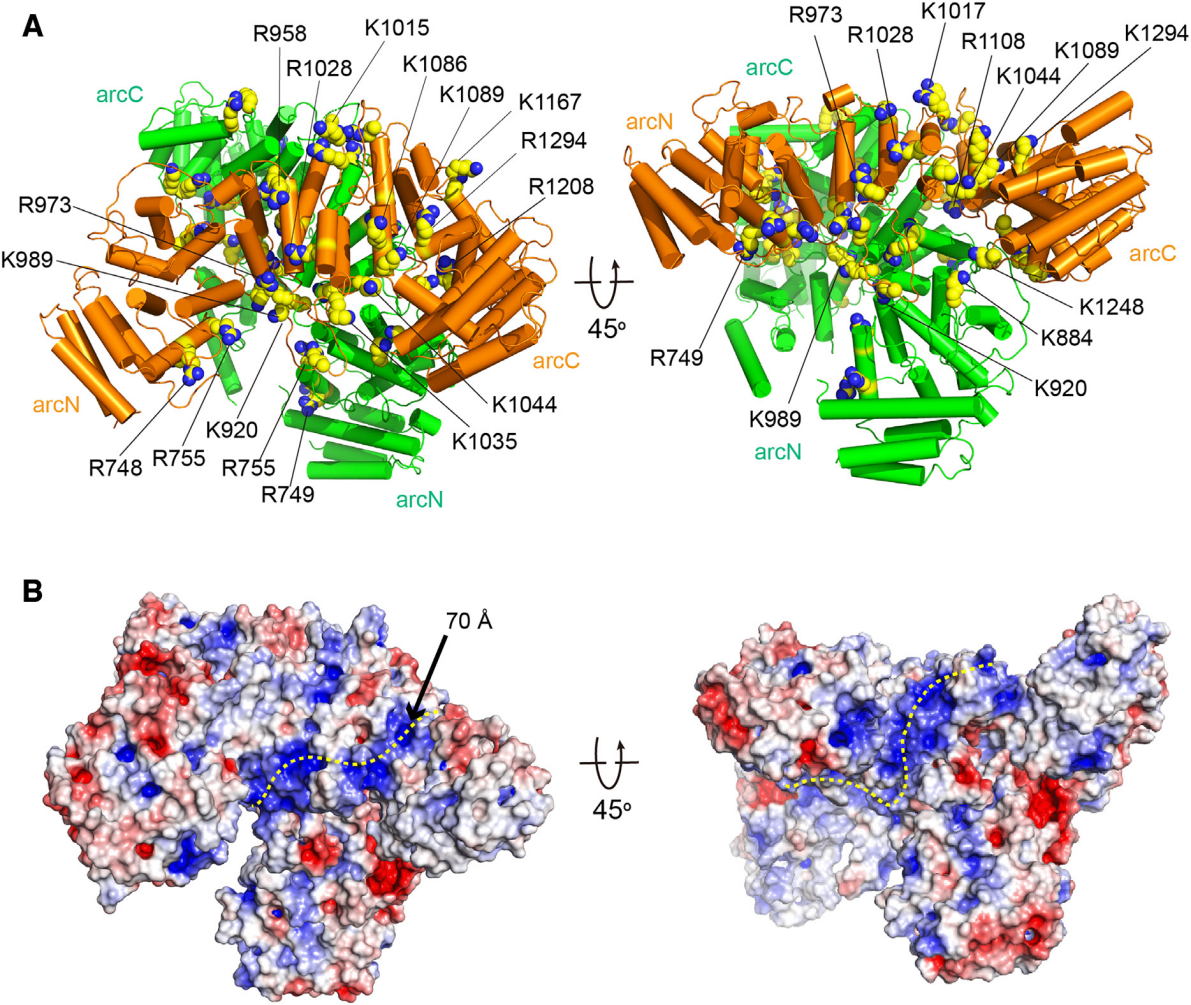
A putative ssDNA binding path in the FANCA CTD dimer. (**A**) Two views of the structure of the FANCA CTD dimer with positively charged residues in space-filling model. (**B**) Surface representation of the FANCA CTD dimer with electrostatic potential in two different views. The electrostatic potential was calculated with Adaptive Poisson-Boltzmann Solver (APBS) and illustrated (–6 to +6 kT) with PyMOL. The positive potential is shown in blue and the negative potential is shown in red. A putative ssDNA binding path is shown in a yellow dotted line. Orientations of the structures are same as those in (A).

Arg1117 is mutated to glycine in a cancer cell line and the mutation of this residue decreases SSA activity of FANCA (28, Supplementary Figure S3). The corresponding residue Lys1089 in *Xl* FANCA is located near the surface. We predict that this residue might interact with ssDNA (Figure 3). Around this site, a narrow groove is formed by the H7B helix, the H8A-H8B loop, the HM3B helix and the C-term loop, through which ssDNA can pass (Figure 3B). Positively charged residues Lys1167, Lys1086, Arg1294, Lys1044, Lys920 and Lys1248 pave the groove along Lys1089 in FANCA CTD (Figure 3A). In addition, the positively charged groove extends continuously to FANCA’ CTD in which Lys1248, Lys989 and Lys920 decorate the positively charged surface. We speculate that the two ssDNA molecules bind to these surface grooves in close proximity in the CTD dimer, providing the basis for the SSA and SSE activities. Importantly, it is also possible that FANCA anchors the core complex near ICLs on chromatin through binding ssDNA regions at stalled replication forks through these positively charged grooves (6).

### Structural basis for the functional defects of patient-derived FANCA mutations

**Figure 4.**
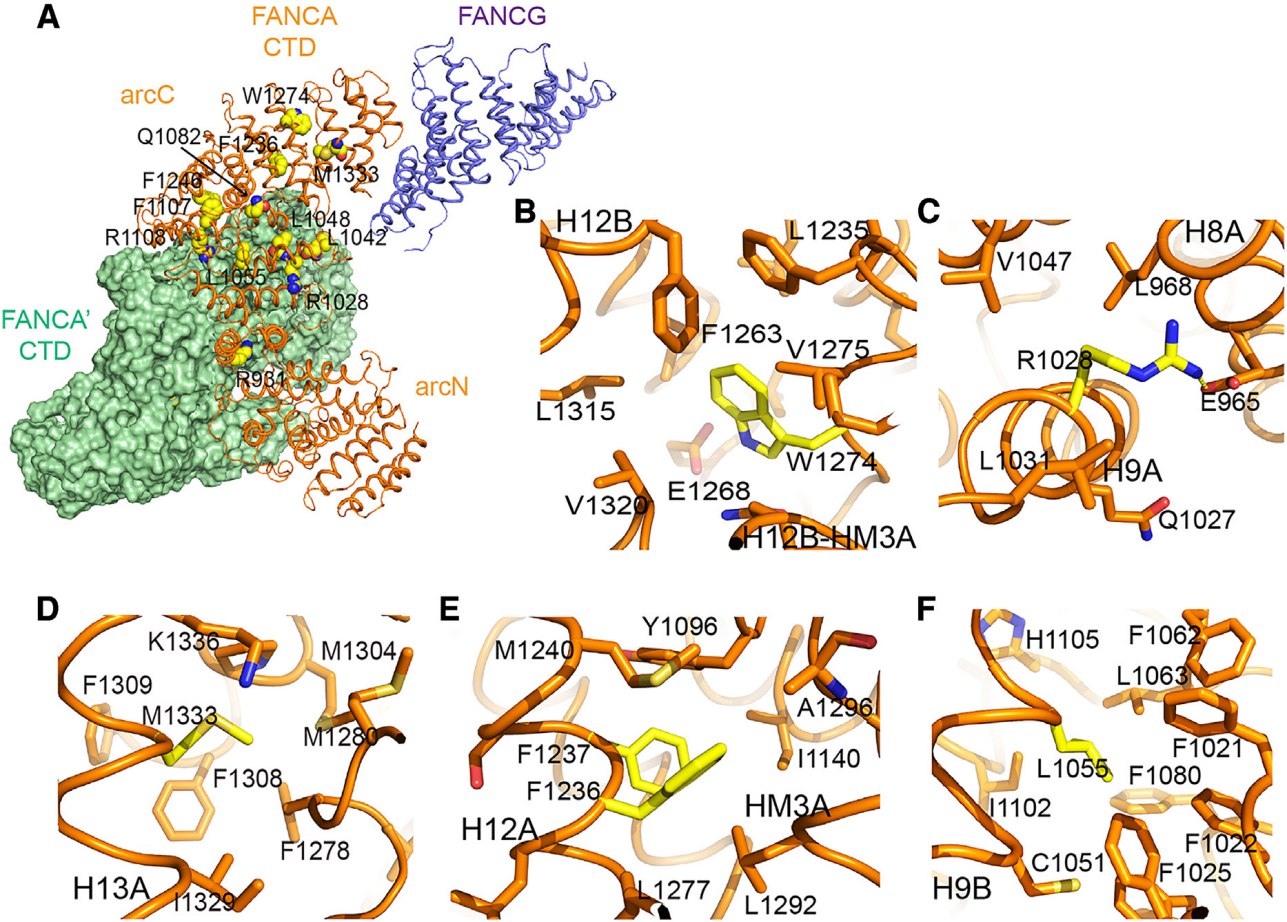
Structure of the selected missense mutations of FANCA from the Fanconi Anemia Mutation Database (http://www2.rockefeller.edu/fanconi/) and Catalogue of Somatic Mutations in Cancer (https://cancer.sanger.ac.uk/cosmic/gene/analysis?ln=FANCA). (**A**) An overall structure of the FANCA CTD complexed with the FANCG CTD (purple). The selected FA- and cancer-associated mutations are illustrated in space filling model. FANCA CTD (green) is shown in surface representation. FANCG is packed against the C-terminal end of a FANCA CTD molecule. (**B**) A mutated residue analyzed in the present study is shown in yellow color. Trp1274 (Trp1302Arg mutation in human FANCA). (**C**) Arg1028 (Arg1055Trp). (**D**) Met1333 (Met1360Ile). (**E**) Phe1236 (Phe1262Leu) and Phe1237 are patient-derived missense and deletion mutation, respectively. (**F**) Leu1055 (Leu1082Pro).

**Figure 5.**
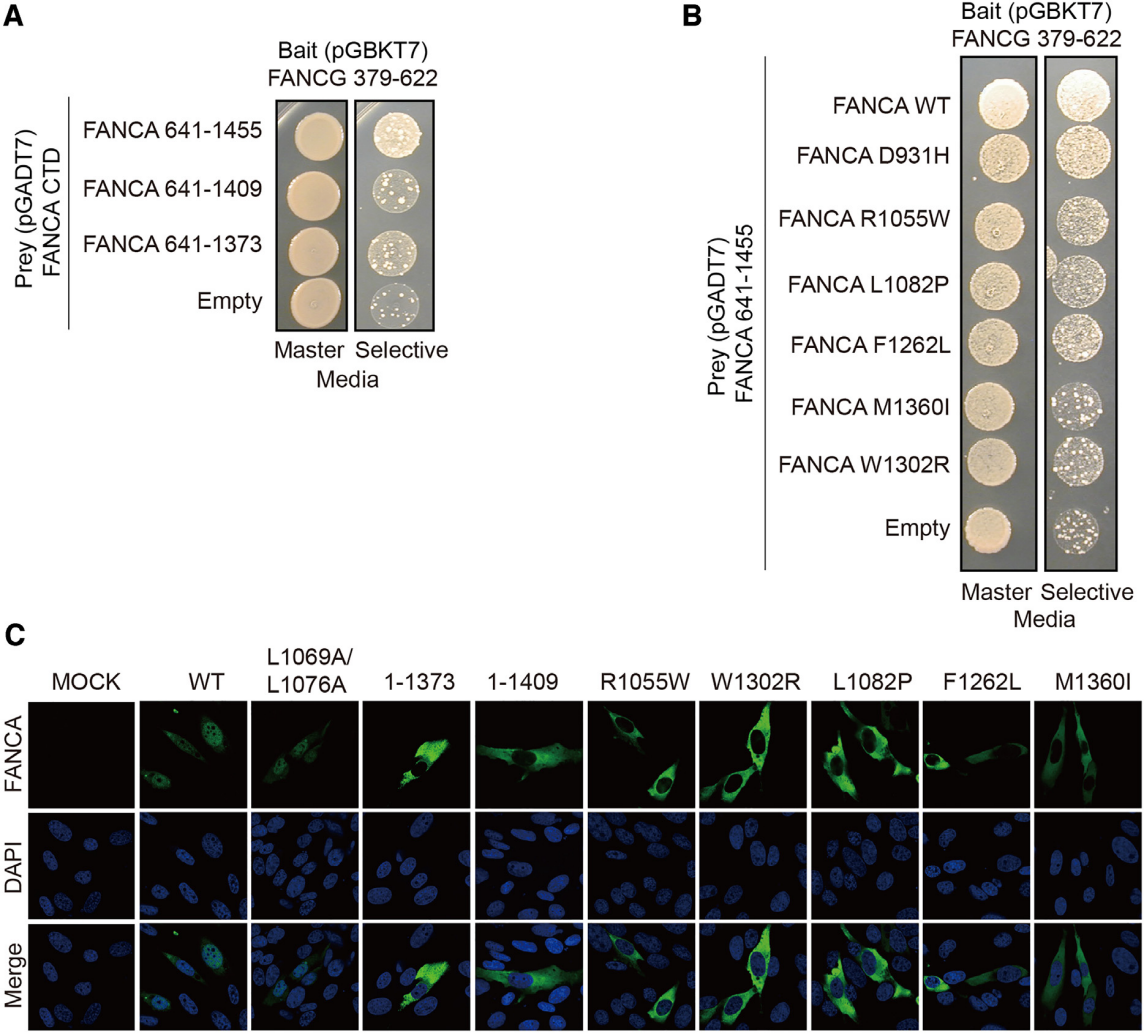
Interactions between FANCA CTD mutants and FANCG CTD and their mutational effects in nuclear localization of FANCA (**A**) Yeast two hybrid analyses between deletion mutants of FANCA CTD and FANCG CTD. Master plate (SD-LeuTrp) and a selective plate (SD-LeuTrpHis containing 10 mM of 3-AT) are shown on the left and right, respectively. (**B**) Yeast two hybrid analyses between FANCA CTD with selected point mutants and FANCG CTD. Master plate (SD-LeuTrp) and a selective plate (SD-LeuTrpHis containing 10 mM of 3-AT) are shown on the left and right, respectively. Structures of the point mutants are shown in Figure 4. (**C**) FANCA-knockout U2OS cells expressing indicated mutants in the presence of 1uM MMC were immunostained to visualize FANCA. Cell nuclei were detected with 4′,6-diamidino-2-phenylindole (DAPI).

FA- and cancer-associated mutations are dispersed over the entire α-solenoid rather than clustered within a localized region. A number of the mutated residues are buried in the interior of the FANCA CTD, indicating that pathogenic effects can result from structural defects in the affected proteins (Figure 4 and Supplementary Figure S6). FANCA mutations lead to mild, intermediate, or severe FA-associated phenotypes (40). In severe FA phenotypes, FANCA mutants fail to interact with FANCC and FANCF, are localized to the cytoplasm, and are unable to monoubiquitinate FANCD2, yet they remain associated with FANCG. Many of the residues that are mutated in severe cases are found to be buried in the core of FANCA, while some are exposed on the surface. For example, Trp1274 (Trp1302Arg mutation in human FANCA) is surrounded by Phe1278, Val1275, Leu1315 and Val1320, and mutation of this residue is predicted to destabilize the local structure (Figure 4B). Arg1028 (the Arg1055Trp mutation in human FANCA) is buried and forms an ion pair with Glu965 in the middle of the arc, and FANCA harboring this mutation fails to be imported into the nucleus (Figures 4C and 5C). However, Gln1082 (the His1110Pro mutation in human FANCA) is exposed at the surface, and it is unlikely that mutation of this residue disrupts the local structure (Supplementary Figure S6D). Nevertheless, mutation of this residue affected interactions with FANCF and FANCC, and the complex failed to localize at the nucleus (40). Thus, Gln1082 is likely to be directly involved in the interaction with other FANC core members.

In addition to point mutations, deletion mutations are also observed in FA patients and several deleted residues are located at the dimeric surface. Deletion of FANCA-Phe1135 or Trp1174 is observed in several FA patients (41,42). Phe1107 (Phe1135 in human FANCA) at the H10B helix of FANCA CTD is located in a hydrophobic environment, packed against the H7B’ helix of FANCA’ CTD (41, Supplementary Figure S6C). Trp1146 (Trp1174 in human FANCA) is at the HM2B-HM2C of FANCA’ CTD, packed against Leu1149, Leu1169, Leu1172 and Arg1173 (42).

Another class of mutants exhibit partially abolished interactions with FANCC or FANCF, moderate nuclear localization, and mild ubiquitination of FANCD2 (40). These mutants include Met1333 (the Met1360Ile mutation in human FANCA) and Tyr796 (the Leu817Pro mutation in human FANCA). Met1333 is surrounded by hydrophobic residues Phe1308, Met1280, Phe1278 and Ile1329 (Figure 4D). Tyr796 is packed against Met750, Leu747, Leu751, Leu800, Leu793 and Trp797 (Supplementary Figure S6E).

The third class of FA- and cancer-associated mutants have no interaction defects with other FANC proteins, and do not exhibit abnormalities in nuclear trafficking or FANCD2 ubiquitination (40). Tyr1100 (the Gln1128Glu mutation in human FANCA) at the dimeric interface belongs to this class (Figure 2C and Supplementary Figure S6C). Thus, disruption of the dimeric interface may not affect binding to the FANC core protein or nuclear trafficking, consistent with the observation described above. Phe1236 (the Phe1262Leu mutation in human FANCA) also belongs to this class. We tested the cellular localization of the Phe1262Leu protein by transiently expressing WT and mutant FANCA protein in U2OS cells and immunostaining after treatment with the crosslinking agent MMC. While the FANCA-WT located to the nucleus as expected, FANCA- Phe1262Leu mutation was localized in cytoplasm in our analyses, consistent with our structural analysis but in contrast to reported data (Figure 5C and Supplementary Table S2). Phe1236 is buried in a hydrophobic environment surrounded by Leu1277, Leu1292, Ile1140 and Met1240, hence its mutation is expected to destabilize the local structure (Figure 4E). We predict that this mutant would exhibit a severe FA phenotype. Mutation of the neighboring Phe1237 residue (delF1263) abrogated the interaction with FANCC and FANCF, and consistent with this observation belongs to the class exhibiting severe phenotype (Figure 4E).

### Interface between FANCA CTD and FANCG CTD is critical for the nuclear import of FANCA

In one class of FANCA and FANCG complex structures, we observed EM density for a twisted helical molecule near the C-terminal end of FANCA. The molecule folds into an extended α-solenoid structure (longest axis with 190 Å) with multiple TPR motifs, and was predicted to correspond to FANCG (43,44). To confirm our prediction, we performed a yeast two-hybrid analysis (Supplementary Figure S7A). We first examined whether FANCG interacts with the CTD of FANCA. In all conditions tested, FANCA CTD unambiguously interacts with the CTD of FANCG. Crosslinking mass spectrometry analysis showed that the CTD of FANCG recognizes the C-terminal end of FANCA (15). Deletion of the C-terminal 37 residues of FANCG resulted in strong reduction in binding to FANCA (45). Thus, based on all available information, we conclude that the molecule corresponding to the EM density is FANCG. We observed EM density for half of the full-length FANCG (319 residues out of 640 residues), and the resolution did not allow us to assign the side-chains. However, the reported biochemical studies (46) and our data described in the next paragraph suggest that the structure corresponds to the C-terminal half of FANCG.

The C-terminal helices (HEAT13 and HEAT14) of the arc^c^ subdomain are packed against the terminal TPR motifs of FANCG that fold into a superhelical structure (Figures 1B and 4A). The convex surface of FANCG forms a shallow groove with three helices from three TPR repeats at the C-terminal end. These helices are packed against the H13 and H14 repeats of FANCA. To confirm our proposed structure, we deleted residues 1374–1455 or residues 1410–1455 at the C-terminal end of FANCA and examined binding to FANCG using yeast-two hybrid analyses. Both deletion mutations significantly decreased binding to FANCA CTD (Figure 5A). In line with this observation, we found that expression of the C-terminal deleted FANCA protein (residues 1–1373 or residues 1–1409) in U2OS yielded exclusively cytoplasmic protein, suggesting that the interface between FANCA CTD and FANCG CTD is important for the nuclear import of FANCA (Figure 5C and Supplementary Table S2).

To explore the significance of this interface in FA and cancer, we mutated five residues of FANCA observed in FA-patients or cancer cells, and examined binding between FANCA CTD and FANCG using yeast two-hybrid analyses. These included Arg1055Trp (Arg1028 in *Xl* FANCA), Leu1082Pro (Leu1055), Phe1262Leu (Phe1236), Trp1302Arg (Trp1274) and Met1360Ile (Met1333). All five FA disease-associated mutants failed to interact with FANCG through their CTDs (Figure 5B). Thus, the residues mutated in these mutants are expected to destabilize the local structure of FANCA. As we observed for the C-terminal deletion mutant of FANCA, expression of all of these five mutants in U2OS cells resulted primarily in cytoplasmic protein in the absence or presence of MMC, consistent with the need for an intact FANCA–FANCG interaction for nuclear transport (Figure 5C and Supplementary Table S2). We conclude that destabilization of the interface between FANCA CTD and FANCG CTD prevents localization of FANCA in the nucleus.

### FANCA recognizes FANCG through its NTD

**Figure 6.**
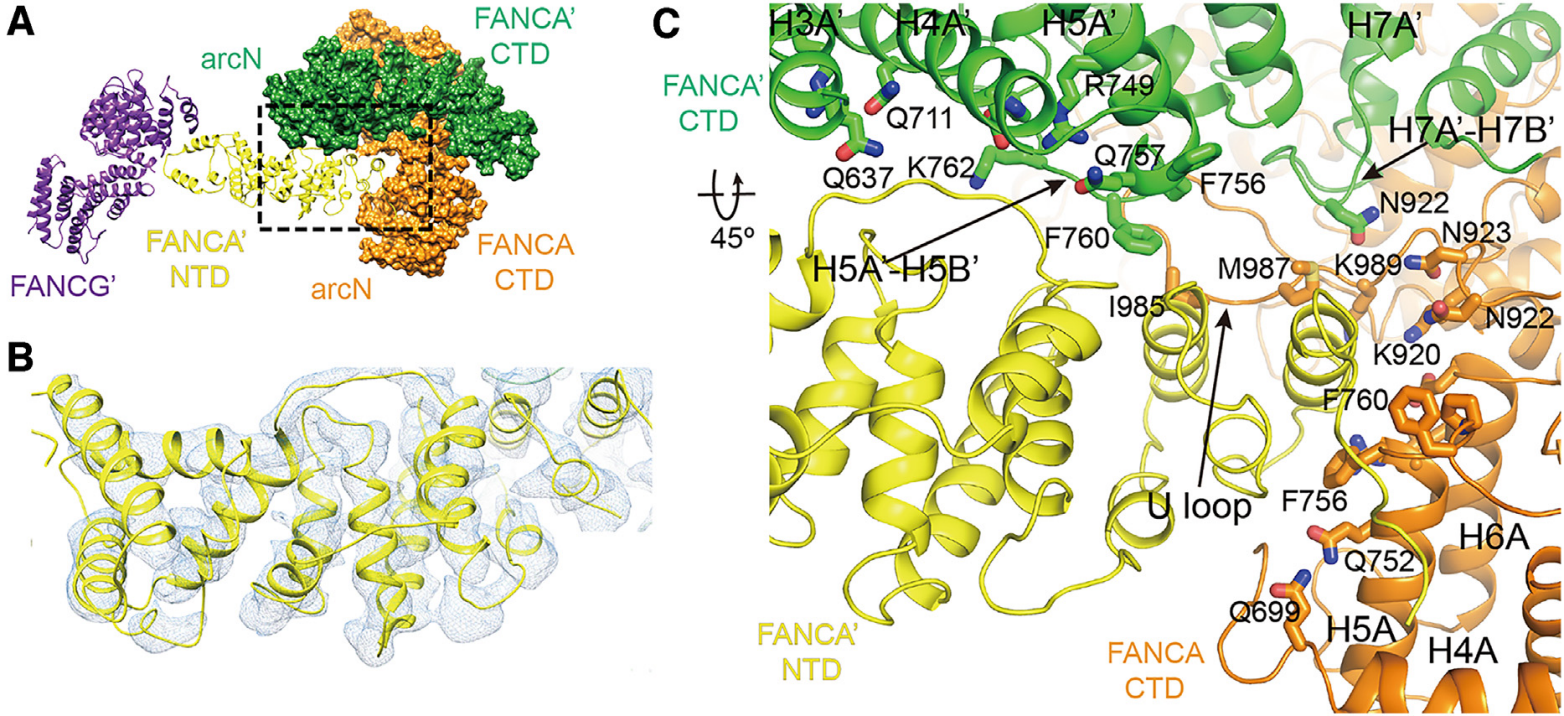
Structure of the FANCA’ complexed with FANCG’ through its NTD. (**A**) The NTD (yellow) of FANCA’ is docked into the inner surface of its CTD dimer. The deep groove is formed at the middle of the inner surface, in which the FANCA’ NTD is lodged. The opposite end of the NTD interacts with FANCG’ (purple). (**B**) The EM density for the NTD of FANCA’. The NTD consists of multiple HEAT repeats. (**C**) Close-up view of the interface between FANCA’ NTD and the CTD dimer. Side chains from the two CTD protomers are shown.

Dimerization of the two FANCA protomers creates continuous concave surface. A long U loop and the H7 helix of FANCA CTD protrude into the middle of this inner surface, forming a deep groove together with arc^N^ subdomains from both FANCA protomers (Figure 6A). The fourth class of structures reveals EM density for an unknown molecule that occupies the groove as well as extensive inner surface of FANCA CTD (Figure 6B). The elongated shaped EM density is formed entirely with helices and with dimensions of 28 × 38 × 93 Å^3^. This unknown molecule could be either FANCG or the NTD of FANCA. We modeled 16 helices onto this density (Figure 6B). However, due to ambiguity, we did not assign side chains in this molecule. The molecule folds into multiple HEAT repeats and contains 310 residues. The molecule interacts with both FANCA and FANCA’. While the groove formed by the U loop and the H5A and the H6A helices from FANCA and the H5A’-H5B’ loop from FANCA’ are mainly involved in the binding to the unknown molecule, the H3A’, H4A’ and H5A’ helices and the H7A’-H7B’ loop from FANCA’ also contribute to the binding (Figure 6C). Thus, both FANCA protomers appear to be important in the recognition of the unknown molecule.

The unknown molecule adopts a significantly different structure from that of FANCG, although we only know the C-terminal half of the FANCG structure. Because the molecule is docked at the concave surface formed by the FANCA and FANCA’ dimer, we hypothesized that the disruption of the FANCA dimeric interface is likely to affect the binding of the molecule to FANCA CTD. We thus generated mutants at or near the dimeric interface of FANCA to examine the effects of the mutation on the binding of FANCG. Specifically, we generated Arg1136Gly (Arg1108 in *Xl* FANCA) mutant, and deleted the U loop spanning Tyr996 to Phe1008 at the concave surface to disrupt the dimeric interface (Figure 2B and C). We also made a Leu1069Ala/Leu1076Ala (Leu1042/Leu1048) double mutant. Previous study showed that the Leu1069Ala/Leu1076Ala mutation dissociates the FANCA dimer into a monomer (28). None of the mutants targeted at the dimeric interface or at the groove affected the interaction between FANCG and FANCA CTD (Figure 5B and Supplementary Figure S7). In addition, we disrupted the groove by introducing Ser1019Arg(Cys999) or Ala1026Arg(Cys1006) mutation at the dimeric interface (Figure 2B). We observed normal interaction between FANCG and these FANCA CTD mutants (Supplementary Figure S7). Crosslinking mass spectrometry analyses revealed a number of intramolecular interactions with the NTD of FANCA in this region (15), supporting our conclusion that the molecule is the NTD of FANCA.

The four helices and the two loops at the C-terminal part of the NTD interact with the CTD dimer. Only the NTD and the helical subdomain of FANCA’, but not that of FANCA is observed inside the arc dimer, suggests that this region of FANCA is flexible and undergoes dynamic rigid body movement. At the N-terminus of NTD, we observed a bulky EM density, which cannot be clearly resolved. The overall shape of FANCG α-solenoid well agrees with this EM density. Previous binding analyses from different groups suggest that the 250 residues of FANCA interact with a region containing 400–475 or the N-terminal 428 residues of FANCG (45,46). The NLS motif containing residues 19–28 of FANCA are critical in this interaction. Our structure shows that the N-terminal region of FANCA binds near the middle rather than the either end of FANCG, consistent with the reported data. The low resolution density for FANCG and the N-terminus of FANCA clearly indicate the dynamic nature of this region in the complex.

### The structure of the FANCA–FANCG hetero-tetramer complex

**Figure 7.**
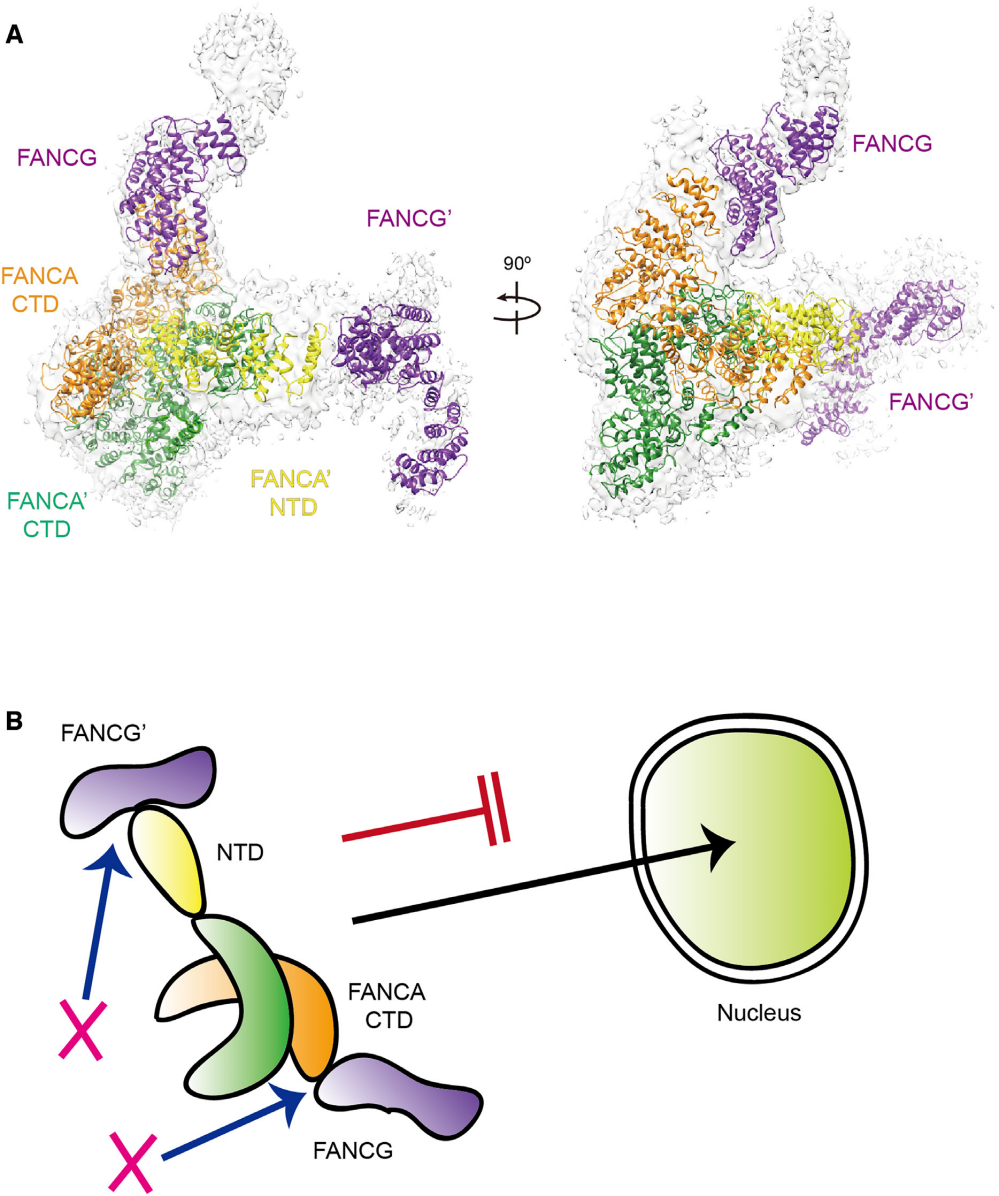
The two interfaces between the FANCA and FANCG complex are required for nuclear transport of FANCA. (**A**) Structure of the FANCA–FANCG hetero-tetramer complex in two different views. The structure was reconstituted by aligning a FANCA CTD dimer of one FANCA–FANCG complex onto that of another complex. The EM map for the two FANCG molecules are shown in gray color. FANCA binds to the FANCG CTD via its C-terminal helical repeats and FANCA’ binds to FANCG’ through its NTD. (**B**) A cartoon representing a model by which disruption of either one of the two interfaces prevents the localization of FANCA into the nucleus.

Although we observed the two different structures of the FANCA and FANCG complex, in which a FANCA homodimer binds to a FANCG protomer at different sites in a 2:1 stoichiometry, the size exclusion analysis showed that FANCA and FANCG forms a 2:2 complex (Supplementary Figure S1). We therefore constructed a model of the hetero-tetramer structure by aligning the FANCA dimer of one AG complex (Figure 1B) onto FANCA’ of another A’G’ complex (Figure 1C). The hetero-tetramer structure forms a curved G-A-A’-G’ chain shape, in which one FANCG molecule is positioned at the end of the NTD of FANCA and another one at the end of FANCA’ CTD, separating the two FANCG protomers by ∼130 Å (Figure 7A). The NTD of FANCA is visible only in one FANCA structure. However, it is possible that the NTD of both FANCA protomers could be visible in the presence of other FANC core components.

The NTD of FANCA undergoes highly dynamic rigid body movement, and the FANCA CTD forms a head to tail dimer. Therefore, in this geometry, it is possible that the two FANCG protomers bind to both ends of one FANCA, in principle, which suggests that the two FANCA protomers can recruit four FANCG protomers. Although we do not completely exclude a possibility that a complex may be formed with different stoichiometry, the hetero-tetramer complex is most consistent with the biochemical data. Furthermore, the presence of other FANC core components may restrict the movement of FANCG and the NTD of FANCA, and thus the stoichiometry of FANCA and FANCG is likely to be limited to 2:2 in the complete core complex. We note that the structure of human FANC core complex containing FANCA and FANCG is reported while this work is under review (47). The structure also reports the two binding modes for FANCA and FANCG in a 2:2 complex—one between FANCA NTD and FANCG and the other between FANCA CTD and FANCG CTD, which supports the present study.

### Putative FAAP20-binding interface within FANCA

FAAP20 binds directly to FANCA and increases its stability (48–51). In the absence of FAAP20, FANCA is exposed and undergoes SUMO-dependent proteolytic degradation, leading to loss of integrity for the FA core complex (52). Mutations that disrupt the binding of FAAP20 exhibit a mild phenotype. Sumoylation occurs at Lys921 (loop HM1C-H7A), equivalent to Arg901 in *Xl* FANCA (Supplementary Figure S8). The exposed Lys903 close to Arg901 is a good candidate for sumoylation. Ile939Ser (Leu919 in *Xl* FANCA) mutation of FANCA exhibits a separation-of-function phenotype since the mutant can assemble the FANCA core complex and monoubiquitinate the ID complex, but it fails to interact and promote translesion DNA synthesis (TLS) polymerization. Leu919 is partly buried in a hydrophobic environment surrounded by Trp915 and Phe970, and 25 Å distant from Lys903. We predict that the mutation of Leu939 to serine likely leads to local structural perturbation (Supplementary Figure S8). One FAAP20 protomer could interact with one FANCA protomer in the AG20 subcomplex, but other FANC core components might affect the stoichiometry between FANCA and FAAP20 in the full FANC core complex.

## DISCUSSION

Over 90% of patient-derived point-mutations in FANCA correspond to residues in the CTD (29). Nevertheless, the structure and function of the FANCA CTD remain poorly understood. In this study, we explore the role of the FANCA CTD in the FA pathway, and provide structural insight into disease-associated FANCA mutations. We present two different structures of the FANCA–FANCG complex as well as structures of FANCA alone. The combined analysis of these two complex structures suggest that two different FANCG protomers bind to two FANCA protomers forming a FANCG–FANCA–FANCA–FANCG hetero-tetramer.

The structures of the FANCA and FANCG complex reveal two important features of the FANCA CTD that explain why it is critical for the function of FANCA and the FANC core complex. The first feature is dimerization of FANCA CTD, which, in addition to a possible role in FA core complex formation, has been suggested to be important for the reported SSA and SSE activities of FANCA (9,12,28). The structure reveals a possible path through which the ssDNA molecule interacts. A long shallow groove is decorated with over 15 positively charged residues in each CTD arc. The groove starts from Lys1167 and Arg1294 at one end and extends to Arg748 at the other end (Figure 3). The distance between the two ends is near 70 Å, indicating that a long ssDNA molecule is required for binding, consistent with the DNA binding analyses. Near the dimeric interface, this groove is connected to the path generated by another FANCA CTD molecule. The grooves from the two FANCA CTDs may therefore locate part of the two ssDNA molecules in close proximity, thereby promoting the SSA activity with the help of FANCA bound to FANCG. While the FANCA CTD dimer provides a binding platform for the two ssDNA molecules, the FANCA NTD complexed with FANCG is required for both SSA and SSE activities (28). Although the mechanism remains unknown, we speculate that dynamic movement of the FANCA NTD complexed with FANCG might increase the chance of the two ssDNA strands encountering each other. Although not yet experimentally explored, the same DNA binding site may also play a role in anchoring FANCA and the core complex on the DNA at stalled replication forks. Because the AG complex contributes to the recruitment of the BL100 complex to the damages sites or chromatin independent of the CEF complex, anchoring of FANCA on the DNA could be critical for the function of FANC core complex (6).

Many FANCA mutations prevent the localization of the protein to the nucleus and cell expressing these mutant FANCA protein are unable to form a nuclear FA core complex. It is unclear if dimerization occurs in the cytosol and is required for FANCA nuclear import. FANCA forms various complexes in the cell; a 0.5 MDa complex in the cytosol, a 2 MDa complex in the nucleus and a 1 MDa complex on chromatin (53). A double mutant L1069A/L1076A that forms a FANCA monomer is efficiently transported into the nucleus (28, Figure 5C). Thus, mutations that affect the dimerization might not be critical for the nuclear localization of FANCA.

Crosslinking mass spectrometry data showed that FANCA interacts with a number of other FANC core proteins including FANCG (15). This network suggests that FANCA undergoes dynamic assembly with other FANC core members. The two different structures of the FANCA and FANCG complexes observed here support the dynamic assembly of FANCA. The observed interaction between FANCG and the FANCA NTD was expected because different groups reported that the NLS of the FANCA NTD is recognized by FANCG (21,24). Deletion of the bipartite NLS motifs either impeded or completely blocked the nuclear localization of FANCA (24,45,54). Two regions of FANCG are involved in the complex formation with FANCA (45). Residues 400–475 are necessary for the binding of FANCG with FANCA and the C-terminal 37 residues (residues 585–622) augment the binding. Due to the limited resolution of the structure for the FANCA NTD and FANCG complex, we were unable to map this interface in atomic detail (Figure 1C). However, the structure suggests that the binding for the N-terminal region of FANCA occurs near the middle region rather than the C-terminal region of FANCA. By contrast, FANCA binding to the FANCG CTD occurring via its helical repeats at the C-terminal end was unexpected. Yeast two-hybrid and crosslinking mass spectrometry analyses confirmed the presence of this interface (15) (Supplementary Figure S7A). Deletion of the C-terminal 37 residues or the two C-terminal helices of FANCA strongly reduced or abrogated the interaction with FANCG, respectively, demonstrating the significance of the interface (45) (Figure 5A). In previous analyses, FA-patient derived mutations exhibiting severe phenotypes retained interaction between FANCA and FANCG (40). Because FANCA binds to FANCG through two distant interfaces (FANCA’ NTD-FANCG’ and FANCA CTD-FANCG CTD), mutations that disrupt one interface are unlikely to affect the other interface, and FANCA still could form a complex with FANCG. Thus, binding of FANCA mutants to FANCG might not reflect all interactions between the two proteins. The five mutants that failed to interact with FANCG through their CTDs were not transported into the nucleus in our analysis (Figure 5B and C; Supplementary Table S2). Thus, we propose that the interaction between FANCA CTD and FANCG CTD is important for nuclear localization. This explains why FANCA mutants lacking the CTD region were able to form a complex with FANCG, but failed to translocate into the nucleus (24).

Overexpression of a FANCA mutant lacking the C-terminal region (residues 1–1200) or the N-terminal 28 residues induced hypersensitivity to MMC (45). Our studies suggest that these results are due to the impaired nuclear localization, emphasizing the importance of the FANCA–FANCG complex formation in nuclear transport of these proteins. FANCG might interact with importin-α and function as an adaptor that mediates the transport of FANCA and other FANC core proteins into the nucleus. Yeast two-hybrid screening identified several candidate proteins that interact with FANCA (55). A set of these proteins are classified as transporters. These include NTF97 (importin beta subunit 97 nuclear transport factor), Sorting nexin 5 and 9 (56), and Kinectin. Alternatively, FANCG itself might function as an importin. Nucleoporin 155 (nuclear pore complex) and Nucleoporin p62, nuclear pore complex directly associated with FANCA in Yeast two-hybrid analyses. Deletion of C-terminal residues of FANCG did not affect the localization of FANCG (46). In our analysis, this is the region that interacts with the C-terminal region of FANCA. Thus, it is possible that FANCG can be translocated to the nucleus independently of FANCA, whereas the nuclear localization of FANCA depends on FANCG. Understanding how the interaction between FANCA CTD and FANCG CTD facilitates nuclear localization requires further investigation.

Although the FANCA–FANCG complex formation via two interfaces is important for nuclear localization of FANCA, forced nuclear targeting of FANCA (residues 29-1455) with SV40 NLS failed to rescue its complementation function (45). This suggests that the formation of the intact G-A-A’-G’ hetero-tetramer structure is not only important for the nuclear transport of FANCA, but also required for the ID monoubiquitination by the FANC core complex. The NTD and the helical subdomain is observed only in one of the FANCA protomers, suggesting that each NTD undergoes significant dynamic motion, which makes FANCA an asymmetric dimer with two different binding sites for FANCG. The asymmetric structure of the FANCA–FANCG hetero-tetramer is compatible with the recently reported FANC core structures with an asymmetric architecture (15,47) In these structures, the FANC core complex is assembled with two BL100 subcomplexes and one CEF complex (PDB ID: 6SRI, see our model in Supplementary Figure S9). The symmetry of the two BL100 hetero-trimers at the center is disrupted by the binding of the CEF substrate-binding module to one side of the two BL100 subcomplexes, which induces a change in orientation in one of the BL100 subcomplexes and prevents the docking of a second CEF on the other side (15,47). Importantly, FANCL in each side of the core complex forms a different conformation, orientation, and packing against nearby components in such a way that only one side of the complex can accommodate the ID substrate, whereas the other side forms an inactive state (15). The structure from Wang *et al.* suggests that only asymmetric FANCA–FANCG hetero-tetramer complex with two different binding modes similar to our structure but not the symmetric complex fits into the two different sides of the FANC core complex (47). Therefore, the asymmetrical G-A-A’-G’ architecture of the FANCA–FANCG complex presented in this study plays an important role in creating the FANCL conformation to accommodate the ID complex only in one side of the complex, thereby monoubiquitinates the ID complex.

The FANCA and FANCG complex structure provides insight into disease-associated FANCA mutations. Several FA- and cancer-associated mutants involve residues at or near the dimeric interface. Most of the mutants at the dimeric interface exhibit significantly decreased SSA or SSE activities, and some of retain normal ID ubiquitination activity (28). Thus, a class of disease-associated mutants that do not or only mildly disrupt the structure at or near the dimeric interface is likely to be associated with defects in these (SSE or SSA) activities, as proposed previously by Zhang's group (28). By contrast, the mutation of buried residues is likely to disrupt the partial or full structure of FANCA in the FA-patients. All five mutants tested herein that belong to this class failed to be imported into the nucleus, which may be largely due to defective interaction with FANCG, but association with other cellular proteins might also contribute to this process.

With only limited biochemical and cellular data available for disease-associated mutants, it is difficult to draw general conclusions pertinent to all patient-derived mutations at present. However, the structure of FANCA coupled with future experimental data on a wider range of FANCA mutants should shed light on FA- and cancer-associated mutations.

## DATA AVAILABILITY

Atomic coordinates and the electron density map for the cryo-EM structure has been deposited in the EM DataBank under accession numbers EMD-0896 (PDB ID: 6LHS), EMD-0899 (PDB ID: 6LHU), EMD-0900 (6LHV) and EMD- EMD-0901 (6LHW).

## Supplementary Material

gkaa062_Supplemental_Files
