# Supplementary material for: Structural basis of the fanconi anemia-associated mutations within the FANCA and FANCG complex
Source: Nucleic Acids Res. 2020 Jan 31;48(6):3328–42. doi: 10.1093/nar/gkaa062 (PMC7102982; doi:10.1093/nar/gkaa062)
Supplement: gkaa062_Supplemental_Files [file gkaa062_supplemental_files.zip › Rev_Supple_DATA_FANCA-.pdf]

**SUPPLEMENTARY DATA**

**SUPPLEMENTARY FIGURE LEGENDS**

Figure S1. Protein purification and workflow of Cryo-EM processing. (A) A profile of size exclusion chromatography and SDS-PAGE analysis of FANCA-FANCG complex. (B) Representative image of a raw micrograph from FANCA and FANCG complex in vitrified ice. (C) Representative image of 2D class averages from reference-free alignment and classification. (D) Flowchart for single particle Cryo-EM processing. Cryo-EM density regions corresponding to each domain, helical subdomain (residues 530-619, red), FANCA NTD (orange) and FANCG (violet), were indicated.

Figure S2. Analysis of cryo-EM data. (A) Local resolution of the FANCA CTD arc. (B) Global resolution of the FANCA CTD arc. (C) Angular distribution of the final reconstruction of the FANCA arc. (D) Local resolution of the FANCA arc with the N-terminal helical subdomain. (E) Global resolution of the FANCA arc with the N-terminal subdomain. (F) Angular distribution of the final reconstruction of the FANCA arc with the N-terminal subdomain. (G) FSC curves between the refined structure and the map calculated from the full dataset (black), the half-map1 (red) and half-map2 (blue). (H) and (I) Two representative cryo-EM density maps at an average resolution of 3.35 Å.

Figure S3. Secondary structure and orthologous conservation of FANCA. The sequence assigned to the FANCA protein is shown. Helices are indicated as cylinders, segments lacking regular secondary structures are represented as solid lines, and disordered regions are illustrated as dotted lines. Residues involved in dimerization are indicated by green squares below the sequence alignment. Mutations identified in the Fanconi Anemia Mutation Database (http://www.rockefeller.edu/fanconi/mutate/) are indicated as red stars, while those identified in the Catalogue of Somatic Mutations in Cancer (https://cancer.sanger.ac.uk/cosmic) are indicated by blue circles. Residues invariant or highly conserved in the six FANCA orthologs are highlighted by yellow and orange bars, respectively. Residues exposed on the surface in one protomer are marked as half-filled circles, and those exposed in both protomers are labeled as empty circles.

Figure S4. Crosslinking analysis of the FANCA and FANCG complex. (A) Addition of 1 and 2 mM BMOE (lane 3 and 4, respectively) to the solution containing the complex induces the oligomerization of the FANCA and FANCG complex. (B) Increasing the concentration of $H_2O_2$ (10, 20, 40, and 80 mM, lane 2 to 5) enhances oligomerization of the FANCA-FANCG complex.

Figure S5. Structural superimposition of FANCA' CTD (green) onto FANCA CTD (orange) by aligning the Cα atoms of all residues of the CTD molecules.

Figure S6. Structures of the selected missense mutations of FANCA from the Fanconi Anemia Mutation Database and Catalogue of Somatic Mutations in Cancer. The selected FA- and cancer-associated mutations are shown in yellow. (A) Leu1042 (Leu1069 in human FANCA) and Leu1048 (Leu1076). (B) Arg931 (Arg951 in human FANCA). (C) Tyr1100 (Gln1128), Phe1107 (Phe1135), and Trp1146 (Trp1174). (D) Gln1082 (His1110). (E) Tyr796 (Leu817)

Figure S7. Yeast two hybrid analyses to examine the interactions between FANCA and FANCG (A) Yeast two hybrid analyses between FANCA and FANCG. The CTD of FANCA clearly interacts with the CTD of FANCG. Master plate (SD-LeuTrp) and a selective plate (SD-LeuTrpHis containing 10 mM of 3-AT) are shown on the left and right, respectively. (B) Yeast two hybrid analyses between FANCA CTD mutants and FANCG CTD. Five mutants including Ser1019Arg, Ala1026Arg, Arg1136Gly, Leu1069Ala/Leu1076Ala and D998-1008 were used for this analysis. All the mutants exhibited normal interactions with FANCG CTD. Master plate (SD-LeuTrp) and a selective plate (SD-LeuTrpHis containing 10 mM of 3-AT) are shown on the left and right, respectively.

Figure S8. The putative FAAP20 binding interface within the FANCA CTD dimer. (A) Overall structure of the FANCA CTD dimer with the FAAP20 binding site marked. Two candidate sites for sumoylation (Arg901 and Lys903) are shown in space filling representation. (B) Close-up view of the FA disease-

associated mutation Leu919 (Ile939Ser in human FANCA), which abrogates the interaction with FAAP20. (C) FA disease-associated missense mutation at Arg859 also disrupts the binding of FANCA to FAAP20.

Figure S9. A model for the FANC core complex. The structure of FANCG of the G-A-A-G' hetero-tetramer complex is aligned with FANCG in the FANC core complex lacking FANCA from the Passmore group (1) (PDB ID 6SRI) to build the model for the full FANC core complex. Asymmetrical structure of the core complex is highlighted by the presence of the CEF subcomplex only in one side of the FANC core complex. FANCL and FANCL' in each side of the complex form different conformations, into which only the asymmetrical AG complex but not the symmetrical one fits. The overall structure is modelled to provide the insights of the asymmetrical architecture of the FANCA-FANCG-complex based on the structure of Wang et al (2). FANCG from the Passmore group (1) is colored in purple, and FANCG from the AG complex is shown in light purple. Two aligned FANCG structures are shown in the right side.

References


1. Shakeel, S., Rajendra, E., Alcon, P., O'Reilly, F., Chorev, D.S., Maslen, S., Degliesposti, G., Russo, C.J., He, S.D., Hill, C.H. *et al.* (2019) Structure of the Fanconi anaemia monoubiquitin ligase complex. *Nature*, **575**, 234-+.
2. Wang, S., Wang, R., Peralta, C., Yaseen, A. and Pavletich, N.P. (2019) Structure of the Fanconi Anemia Core–UBE2T complex poised to ubiquitinate bound FANCI–FANCD2. bioRxiv doi: https://doi.org/10.1101/854158, 25 November 2019, preprint: not peer reviewed.

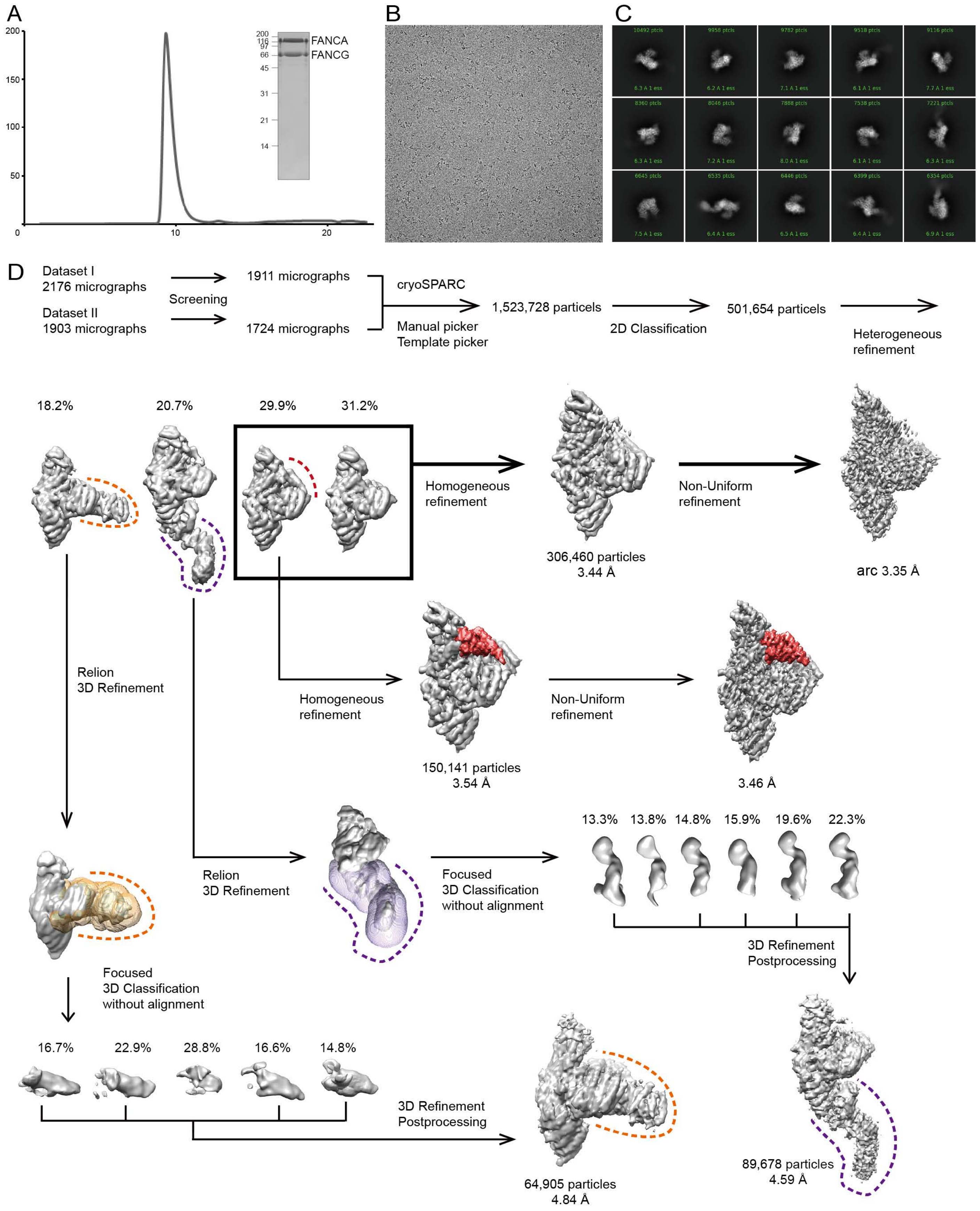


Supplementary Figure S1

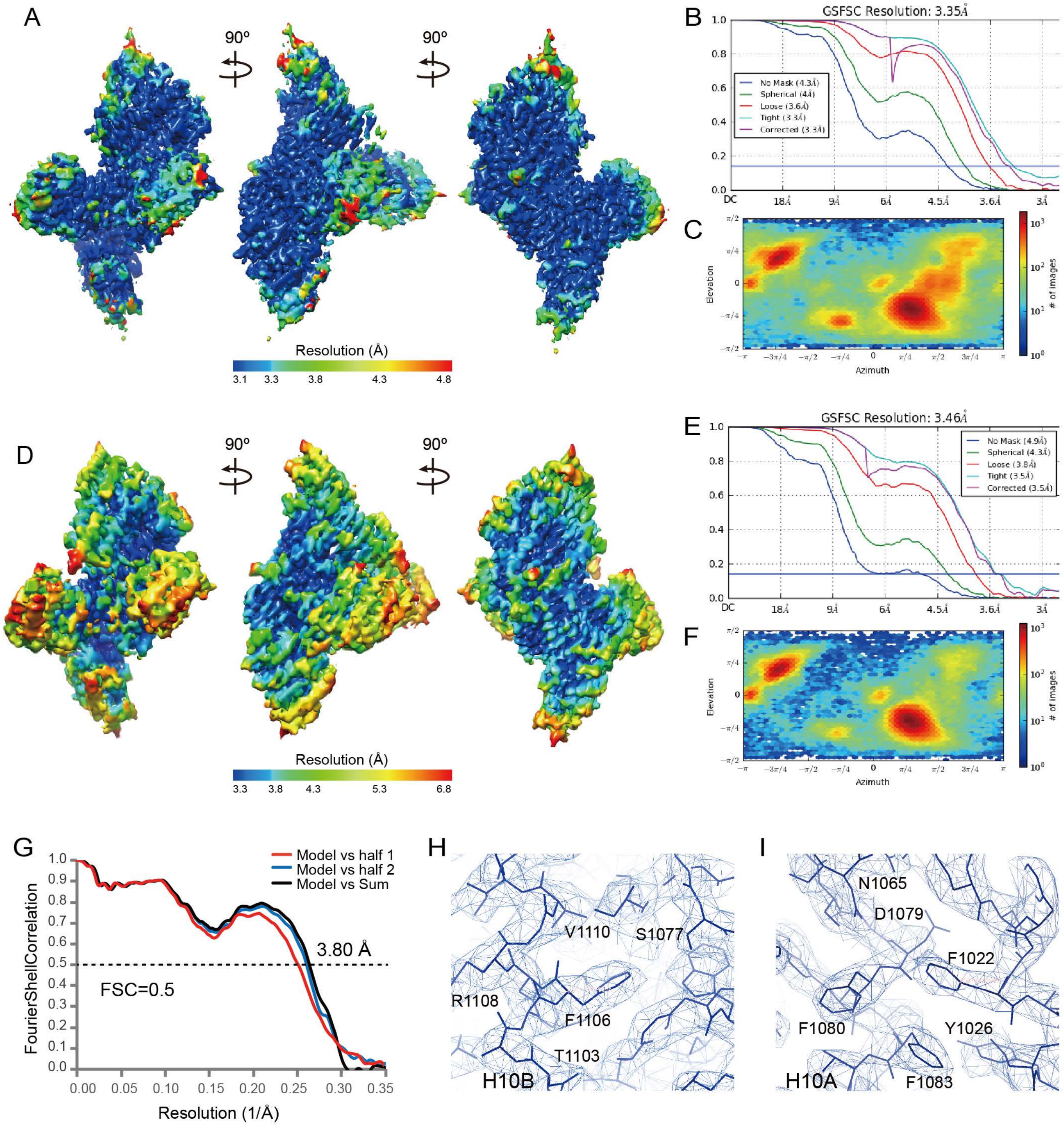


Supplementary Figure S2

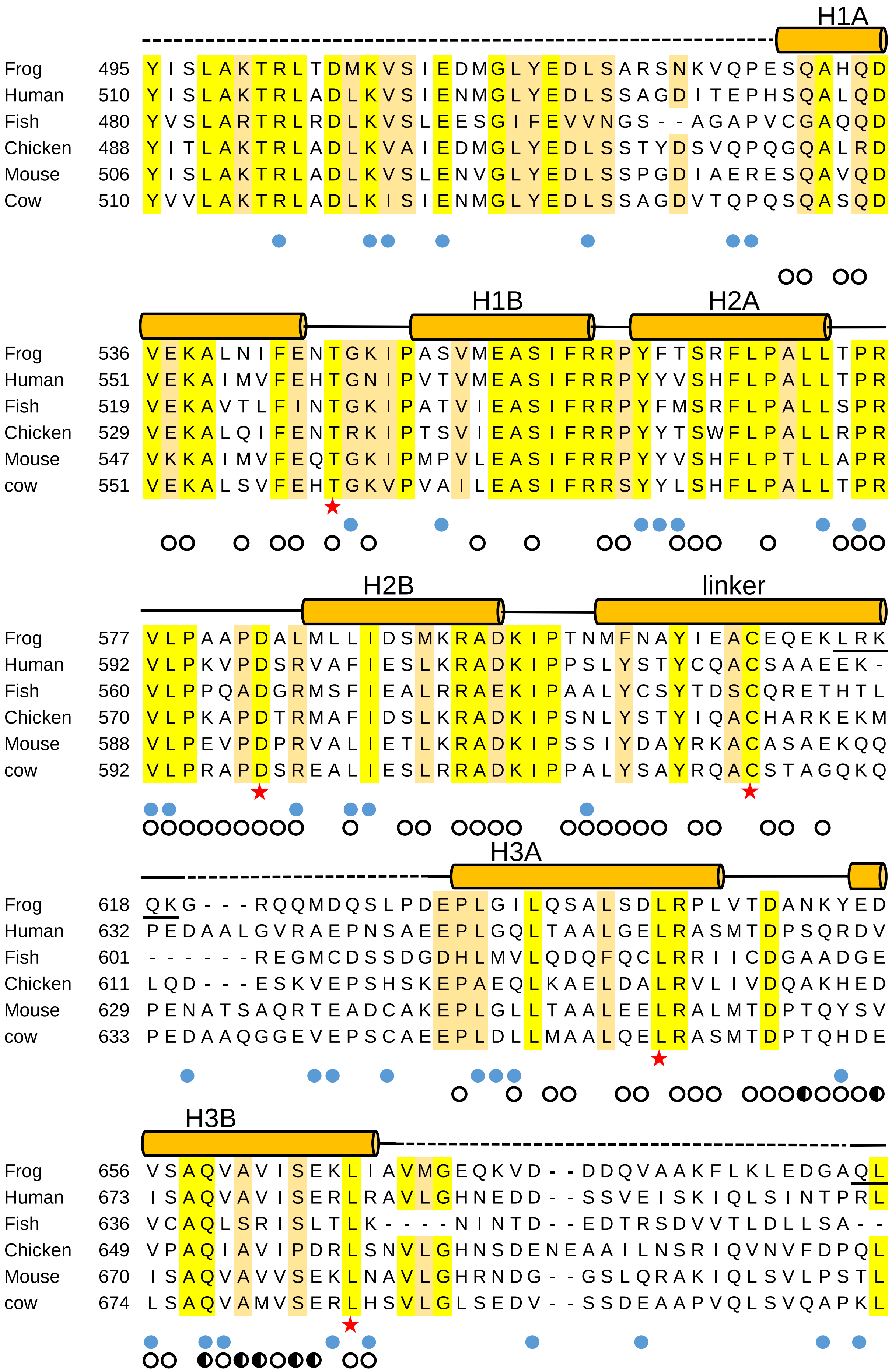
H1A
Frog 495 YISLAKTRLTDMKVSIEDMGLYEDLSARSNKVQPESQAHQD
Human 510 YISLAKTRLADLKVSIENMGLYEDLSSAGDITEPHSQALQD
Fish 480 YVSLARTRLRDLKVSLEESGIFEVVNGS--AGAPVCGAQQD
Chicken 488 YITLAKTRLADLKVAIEDMGLYEDLSSTYDSVQPQGQALRD
Mouse 506 YISLAKTRLADLKVSLENVGLYEDLSSPGDIAERESQAVQD
Cow 510 YVVLAKTRLADLKISIENMGLYEDLSSAGDVTQPQSQASQD
H1B
H2A
Frog 536 VEKALNIFENTGKIPASVMEASIFRRPYFTSRFLPALLTPR
Human 551 VEKAIMVFEHTGNIPVTVMEASIFRRPYYVSHFLPALLTPR
Fish 519 VEKAVTLFINTGKIPATVIEASIFRRPYFMSRFLPALLSPR
Chicken 529 VEKALQIFENTRKIPTSVIEASIFRRPYYTSWFLPALLRPR
Mouse 547 VKKAIMVFEQTGKIPMPVLEASIFRRPYYVSHFLPTLLAPR
cow 551 VEKALSVFEHTGKVPVAILEASIFRRSYYLSHFLPALLTPR
H2B
linker
Frog 577 VLPAAPDALMLLIDSMKRADKIPTNMFNAYIEACEQEKLRK
Human 592 VLPKVPDSRVAFIESLKRADKIPPSLYSTYCQACSAAEEK-
Fish 560 VLPPQADGRMSFIEALRRAEKIPAALYCSYTDSCQRETHTL
Chicken 570 VLPKAPDTRMAFIDSLKRADKIPSNLYSTYIQACHARKEKM
Mouse 588 VLPEVPDPRVALIETLKRADKIPSSIYDAYRKACASAEKQQ
cow 592 VLPRAPDSREALIESLRRADKIPPALYSAYRQACSTAGQKQ
H3A
Frog 618 QKG---RQQMDQSLPDEPLGILQSALSDLRPLVTDANKYED
Human 632 PEDAALGVRAEPNSAEEPLGQLTAALGELRASMTDPSQRDV
Fish 601 ------REGMCDSSDGDHLMVLQDQFQCLRRIICDGAADGE
Chicken 611 LQD---ESKVEPSHSKEPAEQLKAELDALRVLIVDQAKHED
Mouse 629 PENATSAQRTEADCAKEPLGLLTAALEELRALMTDPTQYSV
cow 633 PEDAAQGGEVEPSCAEEPLDLLMAALQELRASMTDPTQHDE
H3B
Frog 656 VSAQVAVISEKLIAVMGEQKVD--DDQVAAKFLKLEDGAQL
Human 673 ISAQVAVISERLRAVLGHNEDD--SSVEISKIQLSINTPRL
Fish 636 VCAQLSRISLTLK----NINTD--EDTRSDVVTLDLLSA--
Chicken 649 VPAQIAVIPDRLSNVLGHNSDENEAAILNSRIQVNVFDPQL
Mouse 670 ISAQVAVVSEKLNAVLGHRNDG--GSLQRAKIQLSVLPSTL
cow 674 LSAQVAMVSERLHSVLGLSEDV--SSDEAAPVQLSVQAPKL

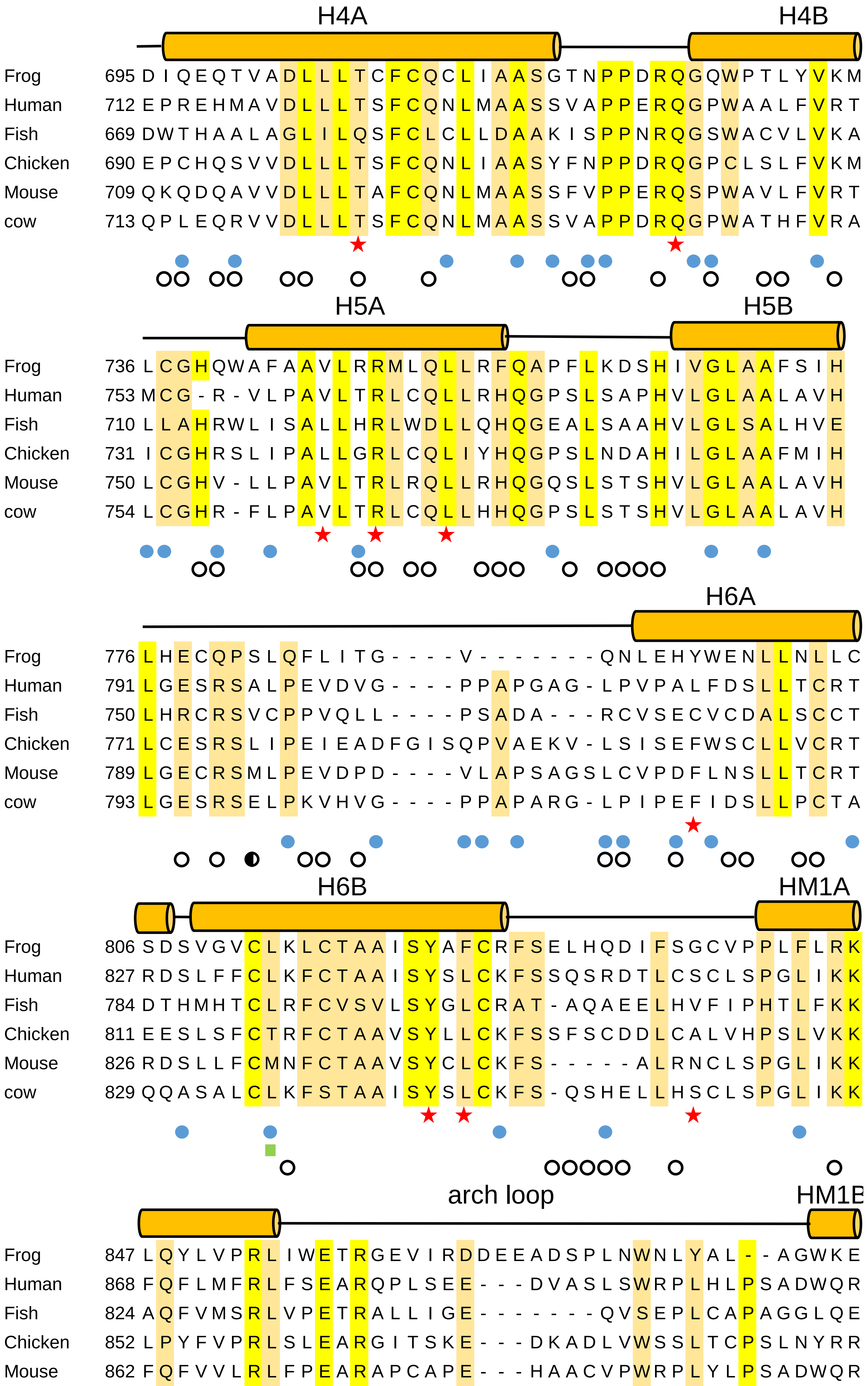
H4A
H4B
Frog 695 D I Q E Q T V A D L L L T C F C Q C L I A A S G T N P P D R Q G Q W P T L Y V K M
Human 712 E P R E H M A V D L L L T S F C Q N L M A A S S V A P P E R Q G P W A A L F V R T
Fish 669 D W T H A A L A G L I L Q S F C L C L L D A A K I S P P N R Q G S W A C V L V K A
Chicken 690 E P C H Q S V V D L L L T S F C Q N L I A A S Y F N P P D R Q G P C L S L F V K M
Mouse 709 Q K Q D Q A V V D L L L T A F C Q N L M A A S S F V P P E R Q S P W A V L F V R T
cow 713 Q P L E Q R V V D L L L T S F C Q N L M A A S S V A P P D R Q G P W A T H F V R A
H5A
H5B
Frog 736 L C G H Q W A F A A V L R R M L Q L L R F Q A P F L K D S H I V G L A A F S I H
Human 753 M C G - R - V L P A V L T R L C Q L L R H Q G P S L S A P H V L G L A A L A V H
Fish 710 L L A H R W L I S A L L H R L W D L L Q H Q G E A L S A A H V L G L S A L H V E
Chicken 731 I C G H R S L I P A L L G R L C Q L I Y H Q G P S L N D A H I L G L A A F M I H
Mouse 750 L C G H V - L L P A V L T R L R Q L L R H Q G Q S L S T S H V L G L A A L A V H
cow 754 L C G H R - F L P A V L T R L C Q L L H H Q G P S L S T S H V L G L A A L A V H
H6A
Frog 776 L H E C Q P S L Q F L I T G - - - - V - - - - - - - Q N L E H Y W E N L L N L C
Human 791 L G E S R S A L P E V D V G - - - - P P A P G A G - L P V P A L F D S L L T C R T
Fish 750 L H R C R S V C P P V Q L L - - - - P S A D A - - - R C V S E C V C D A L S C C T
Chicken 771 L C E S R S L I P E I E A D F G I S Q P V A E K V - L S I S E F W S C L L V C R T
Mouse 789 L G E C R S M L P E V D P D - - - - V L A P S A G S L C V P D F L N S L L T C R T
cow 793 L G E S R S E L P K V H V G - - - - P P A P A R G - L P I P E F I D S L L P C T A
H6B
HM1A
Frog 806 S D S V G V C L K L C T A A I S Y A F C R F S E L H Q D I F S G C V P P L F L R K
Human 827 R D S L F F C L K F C T A A I S Y S L C K F S S Q S R D T L C S C L S P G L I K K
Fish 784 D T H M H T C L R F C V S V L S Y G L C R A T - A Q A E E L H V F I P H T L F K K
Chicken 811 E E S L S F C T R F C T A A V S Y L L C K F S S F S C D D L C A L V H P S L V K K
Mouse 826 R D S L L F C M N F C T A A V S Y C L C K F S - - - - - A L R N C L S P G L I K K
cow 829 Q Q A S A L C L K F S T A A I S Y S L C K F S - Q S H E L L H S C L S P G L I K K
arch loop
Frog 847 L Q Y L V P R L I W E T R G E V I R D D E E A D S P L N W N L Y A L - - A G W K E
Human 868 F Q F L M F R L F S E A R Q P L S E E - - - D V A S L S W R P L H L P S A D W Q R
Fish 824 A Q F V M S R L V P E T R A L L I G E - - - - - - - - Q V S E P L C A P A G G L Q E
Chicken 852 L P Y F V P R L S L E A R G I T S K E - - - D K A D L V W S S L T C P S L N Y R R
Mouse 862 F Q F V V L R L F P E A R A P C A P E - - - H A A C V P W R P L Y L P S A D W Q R

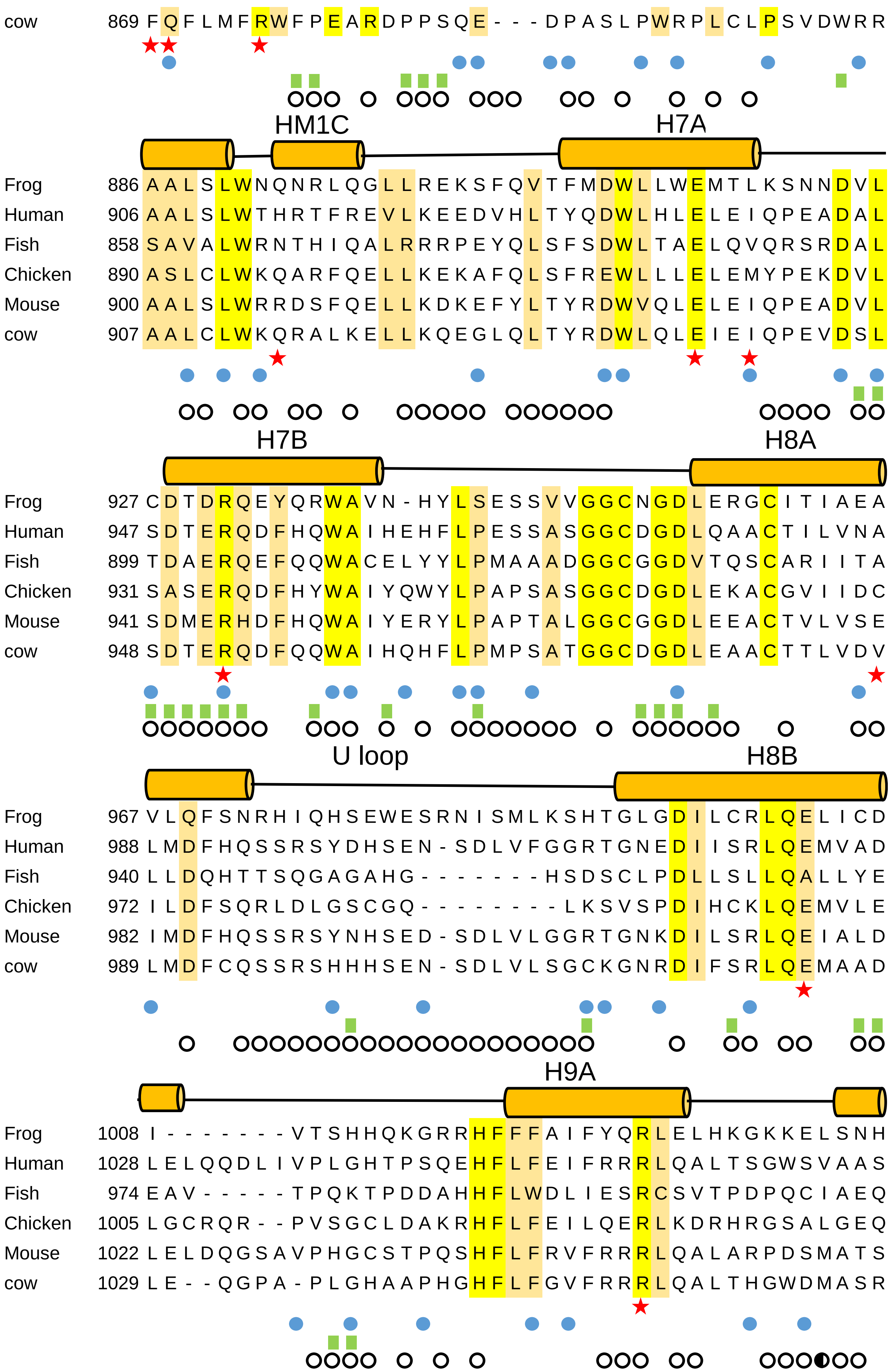

cow 869 FQFLMFRWFPEARDPPSQE---DPASLPWRPLCLPSVDWRR
HM1C
H7A
Frog 886 AALSLWNQNRLQGLLREKSFQVTFMDWLLWEMTLKSNNDVL
Human 906 AALSLWTHRTFREVLKEEDVHLTYQDWLHLELEIQPEADAL
Fish 858 SAVALWRNTHIQALRRRPEYQLSFSDWLTAELQVQRSRDAL
Chicken 890 ASLCLWKQARFQELLKEKAFQLSFREWLLLELEMYPEKDVL
Mouse 900 AALSLWRRDSFQELLKDKEFYLTYRDWVQLELEIQPEADVL
cow 907 AALCLWKQRALKELLKQEGLQLTYRDWLQLEIEIQPEVDSL
H7B
H8A
Frog 927 CDTDRQEYQRWAVN-HYLSESSVVGGCNGDLERGCITIAEA
Human 947 SDTERQDFHQWAIHEHFLPESSASGGCDGDLQAACTILVNA
Fish 899 TDAERQEFQQWACELYYLPMAAADGGCGGDVTQSCARIITA
Chicken 931 SASERQDFHYWAIYQWYLPAPSASGGCDGDLEKACGVIIDC
Mouse 941 SDMERHDFHQWAIYERYLPAPTALGGCGGDLEEACTVLVSE
cow 948 SDTERQDFQQWAIHQHFLPMPSATGGCDGDLEAACTTLVDV
U loop
H8B
Frog 967 VLQFSNRHIQHSEWESRNISMLKSHTGLGDILCRLQELICD
Human 988 LMDFHQSSRSYDHSEN-SDLVFGGRTGNEDIISRLQEMVAD
Fish 940 LLDQHTTSQGAGAHG-------HSDSCLPDLLSLLQALLYE
Chicken 972 ILDFSQRLDLGSCGQ--------LKSVSPDIHCKLQEMVLE
Mouse 982 IMDFHQSSRSYNHSED-SDLVLGGRTGNKDILSRLQEIALD
cow 989 LMDFCQSSRSHHHSEN-SDLVLSGCKGNRDIFSRLQEMAAD
H9A
Frog 1008 I-------VTSHHQKGRRHFFFAIFYQRLELHKGKKELSNH
Human 1028 LELQQDLIVPLGHTPSQEHFLFEIFRRRLQALTSGWSVAAS
Fish 974 EAV-----TPQKTPDDAHHFLWDLIESRCSVTPDPQCIAEQ
Chicken 1005 LGCRQR--PVSGCLDAKRHFLFEILQERLKDRHRGSALGEQ
Mouse 1022 LELDQGSAVPHGCSTPQSHFLFRVFRRRLQALARPDSMATS
cow 1029 LE--QGPA-PLGHAAPHGHFLFGVFRRRLQALTHGWDMASR

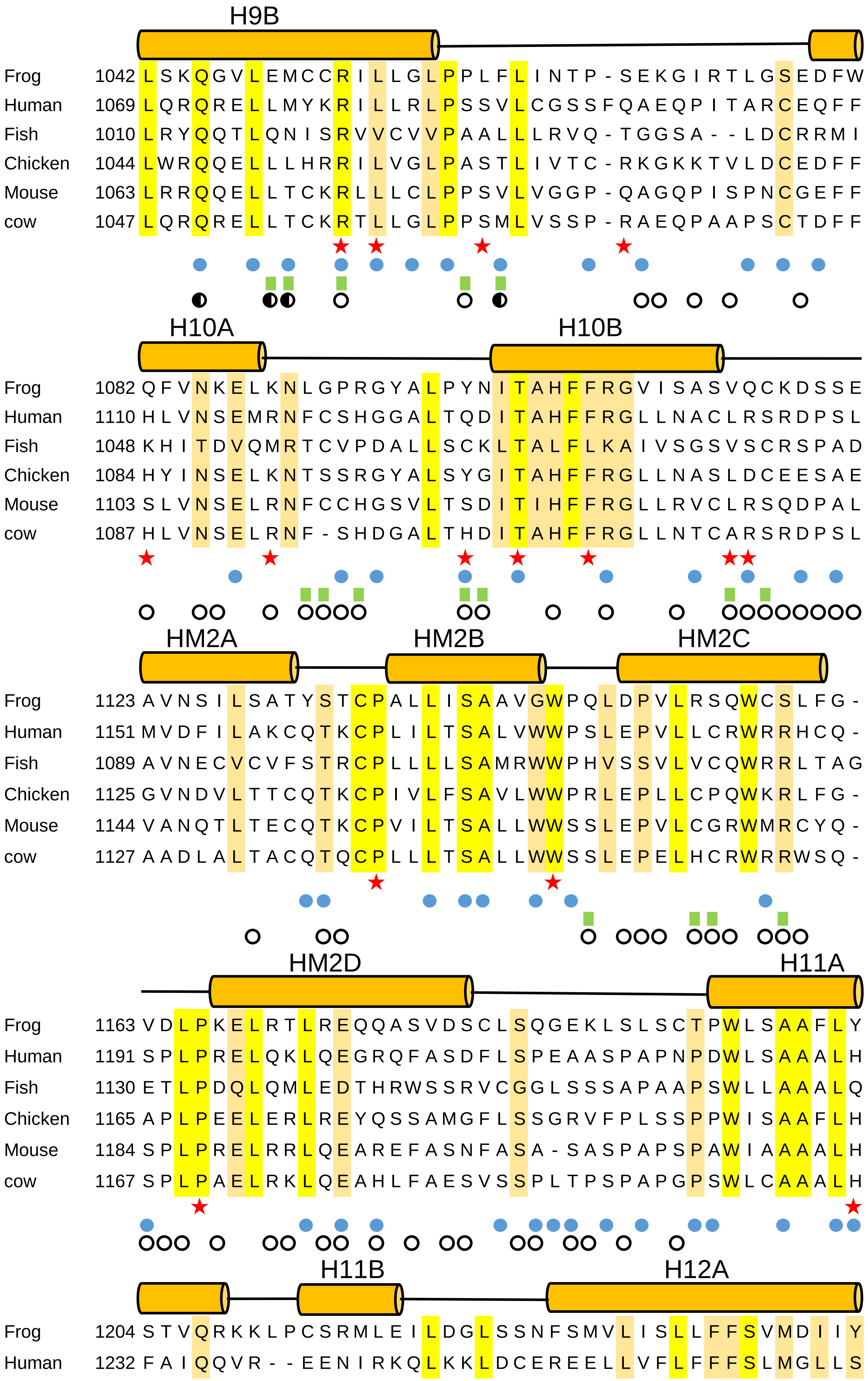
H9B
Frog 1042 L S K Q G V L E M C C R I L L G L P P L F L I N T P - S E K G I R T L G S E D F W
Human 1069 L Q R Q R E L L M Y K R I L L R L P S S V L C G S S F Q A E Q P I T A R C E Q F F
Fish 1010 L R Y Q Q T L Q N I S R V V C V V P A A L L L R V Q - T G G S A - - L D C R R M I
Chicken 1044 L W R Q Q E L L L H R R I L V G L P A S T L I V T C - R K G K K T V L D C E D F F
Mouse 1063 L R R Q Q E L L T C K R L L L C L P P S V L V G G P - Q A G Q P I S P N C G E F F
cow 1047 L Q R Q R E L L T C K R T L L G L P P S M L V S S P - R A E Q P A A P S C T D F F
H10A
H10B
Frog 1082 Q F V N K E L K N L G P R G Y A L P Y N I T A H F F R G V I S A S V Q C K D S S E
Human 1110 H L V N S E M R N F C S H G G A L T Q D I T A H F F R G L L N A C L R S R D P S L
Fish 1048 K H I T D V Q M R T C V P D A L L S C K L T A L F L K A I V S G S V S C R S P A D
Chicken 1084 H Y I N S E L K N T S S R G Y A L S Y G I T A H F F R G L L N A S L D C E E S A E
Mouse 1103 S L V N S E L R N F C C H G S V L T S D I T I H F F R G L L R V C L R S Q D P A L
cow 1087 H L V N S E L R N F - S H D G A L T H D I T A H F F R G L L N T C A R S R D P S L
HM2A
HM2B
HM2C
Frog 1123 A V N S I L S A T Y S T C P A L L I S A A V G W P Q L D P V L R S Q W C S L F G -
Human 1151 M V D F I L A K C Q T K C P L I L T S A L V W W P S L E P V L L C R W R R H C Q -
Fish 1089 A V N E C V C V F S T R C P L L L L S A M R W W P H V S S V L V C Q W R R L T A G
Chicken 1125 G V N D V L T T C Q T K C P I V L F S A V L W W P R L E P L L C P Q W K R L F G -
Mouse 1144 V A N Q T L T E C Q T K C P V I L T S A L L W W S S L E P V L C G R W M R C Y Q -
cow 1127 A A D L A L T A C Q T Q C P L L L T S A L L W W S S L E P E L H C R W R R W S Q -
HM2D
H11A
Frog 1163 V D L P K E L R T L R E Q Q A S V D S C L S Q G E K L S L S C T P W L S A A F L Y
Human 1191 S P L P R E L Q K L Q E G R Q F A S D F L S P E A A S P A P N P D W L S A A A L H
Fish 1130 E T L P D Q L Q M L E D T H R W S S R V C G G L S S S A P A A P S W L L A A A L Q
Chicken 1165 A P L P E E L E R L R E Y Q S S A M G F L S S G R V F P L S S P P W I S A A F L H
Mouse 1184 S P L P R E L R R L Q E A R E F A S N F A S A - S A S P A P S P A W I A A A A L H
cow 1167 S P L P A E L R K L Q E A H L F A E S V S S P L T P S P A P G P S W L C A A A L H
H11B
H12A
Frog 1204 S T V Q R K K L P C S R M L E I L D G L S S N F S M V L I S L L F F S V M D I I Y
Human 1232 F A I Q Q V R - - E E N I R K Q L K K L D C E R E E L L V F L F F F S L M G L L S

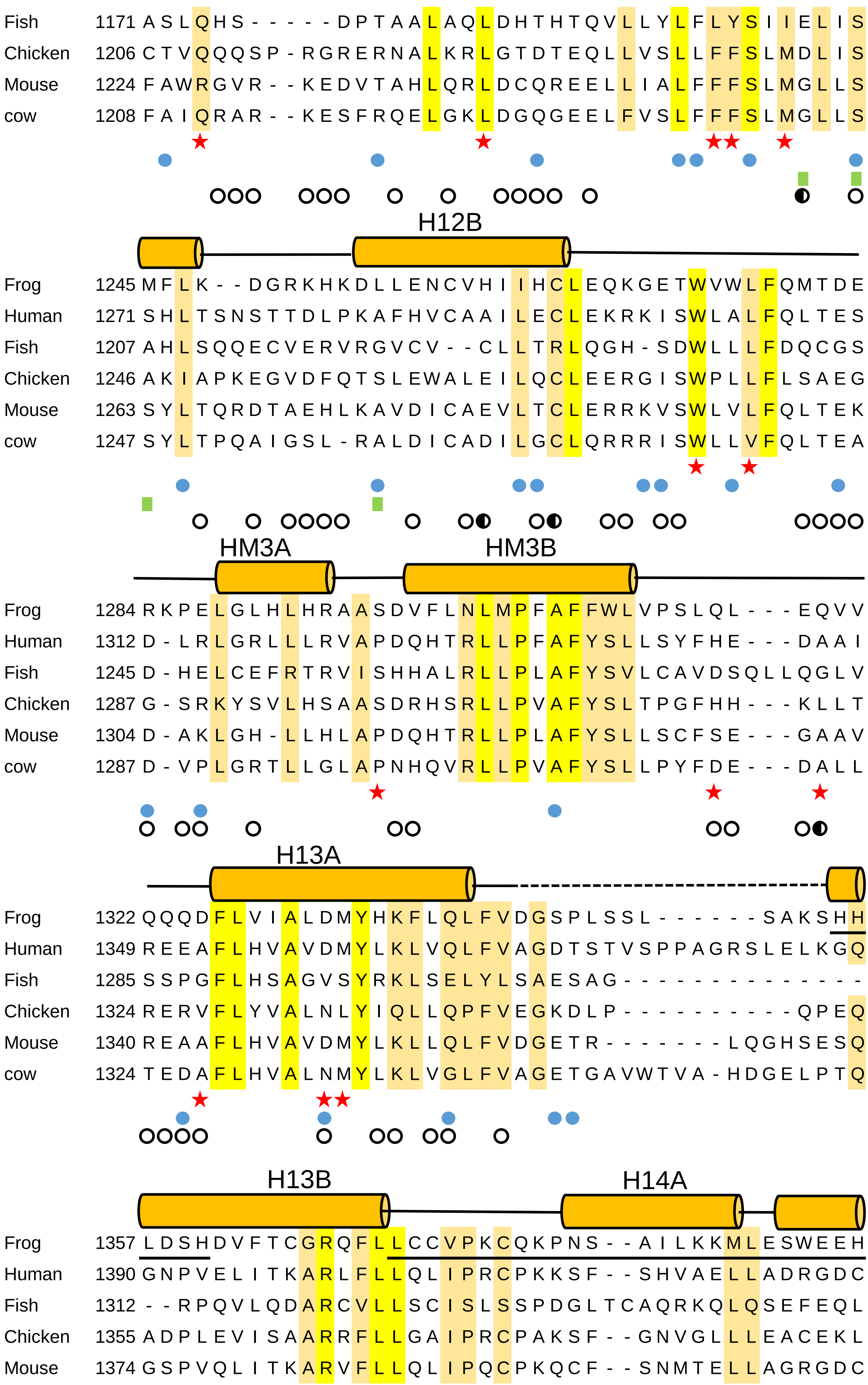
Fish 1171 A S L Q H S - - - - - D P T A A L A Q L D H T H T Q V L L Y L F L Y S I I E L I S
Chicken 1206 C T V Q Q Q S P - R G R E R N A L K R L G T D T E Q L L V S L L F F S L M D L I S
Mouse 1224 F A W R G V R - - K E D V T A H L Q R L D C Q R E E L L I A L F F F S L M G L L S
cow 1208 F A I Q R A R - - K E S F R Q E L G K L D G Q G E E L F V S L F F F S L M G L L S
H12B
Frog 1245 M F L K - - D G R K H K D L L E N C V H I I H C L E Q K G E T W V W L F Q M T D E
Human 1271 S H L T S N S T T D L P K A F H V C A A I L E C L E K R K I S W L A L F Q L T E S
Fish 1207 A H L S Q Q E C V E R V R G V C V - - C L L T R L Q G H - S D W L L L F D Q C G S
Chicken 1246 A K I A P K E G V D F Q T S L E W A L E I L Q C L E E R G I S W P L L F L S A E G
Mouse 1263 S Y L T Q R D T A E H L K A V D I C A E V L T C L E R R K V S W L V L F Q L T E K
cow 1247 S Y L T P Q A I G S L - R A L D I C A D I L G C L Q R R R I S W L L V F Q L T E A
HM3A
HM3B
Frog 1284 R K P E L G L H L H R A A S D V F L N L M P F A F F W L V P S L Q L - - - E Q V V
Human 1312 D - L R L G R L L L R V A P D Q H T R L L P F A F Y S L L S Y F H E - - - D A A I
Fish 1245 D - H E L C E F R T R V I S H H A L R L L P L A F Y S V L C A V D S Q L L Q G L V
Chicken 1287 G - S R K Y S V L H S A A S D R H S R L L P V A F Y S L T P G F H H - - - K L L T
Mouse 1304 D - A K L G H - L L H L A P D Q H T R L L P L A F Y S L L S C F S E - - - G A A V
cow 1287 D - V P L G R T L L G L A P N H Q V R L L P V A F Y S L L P Y F D E - - - D A L L
H13A
Frog 1322 Q Q Q D F L V I A L D M Y H K F L Q L F V D G S P L S S L - - - - - - S A K S H H
Human 1349 R E E A F L H V A V D M Y L K L V Q L F V A G D T S T V S P P A G R S L E L K G Q
Fish 1285 S S P G F L H S A G V S Y R K L S E L Y L S A E S A G - - - - - - - - - - - - - -
Chicken 1324 R E R V F L Y V A L N L Y I Q L L Q P F V E G K D L P - - - - - - - - - - Q P E Q
Mouse 1340 R E A A F L H V A V D M Y L K L L Q L F V D G E T R - - - - - - - L Q G H S E S Q
cow 1324 T E D A F L H V A L N M Y L K L V G L F V A G E T G A V W T V A - H D G E L P T Q
H13B
H14A
Frog 1357 L D S H D V F T C G R Q F L L C C V P K C Q K P N S - - A I L K K M L E S W E E H
Human 1390 G N P V E L I T K A R L F L L Q L I P R C P K K S F - - S H V A E L L A D R G D C
Fish 1312 - - R P Q V L Q D A R C V L L S C I S L S S P D G L T C A Q R K Q L Q S E F E Q L
Chicken 1355 A D P L E V I S A A R R F L L G A I P R C P A K S F - - G N V G L L L E A C E K L
Mouse 1374 G S P V Q L I T K A R V F L L Q L I P Q C P K Q C F - - S N M T E L L A G R G D C

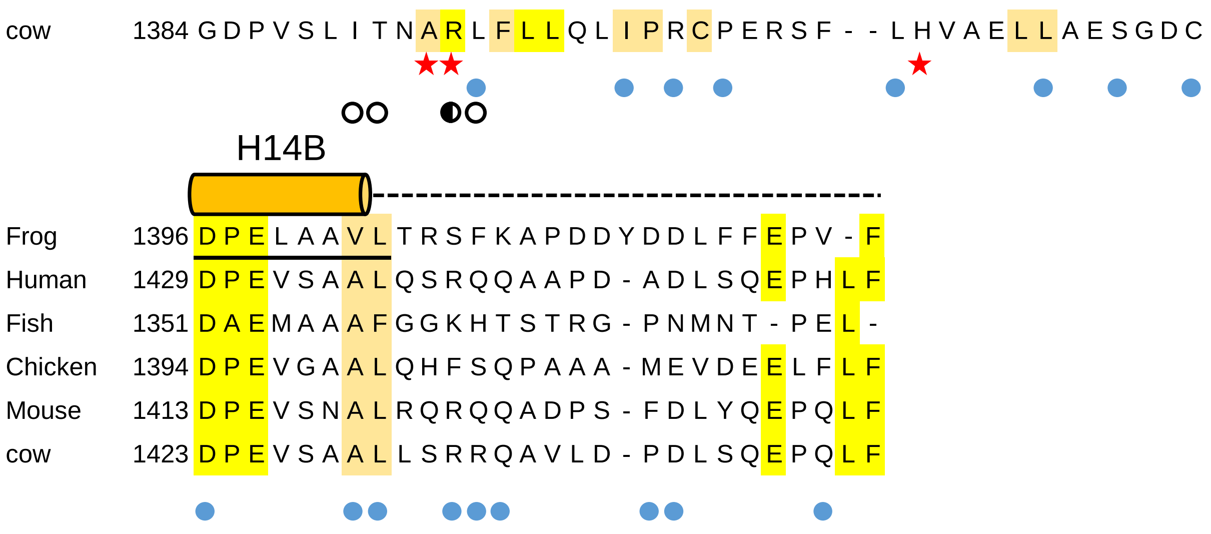


Supplementary Figure S3

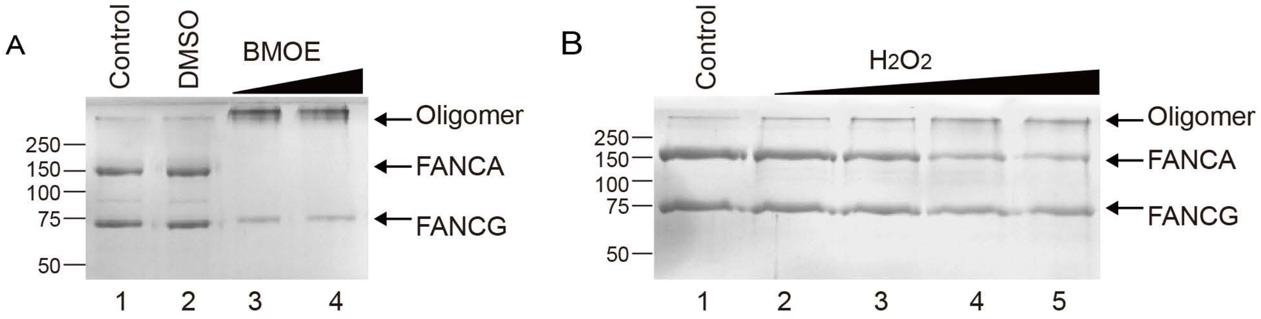


Supplementary Figure S4

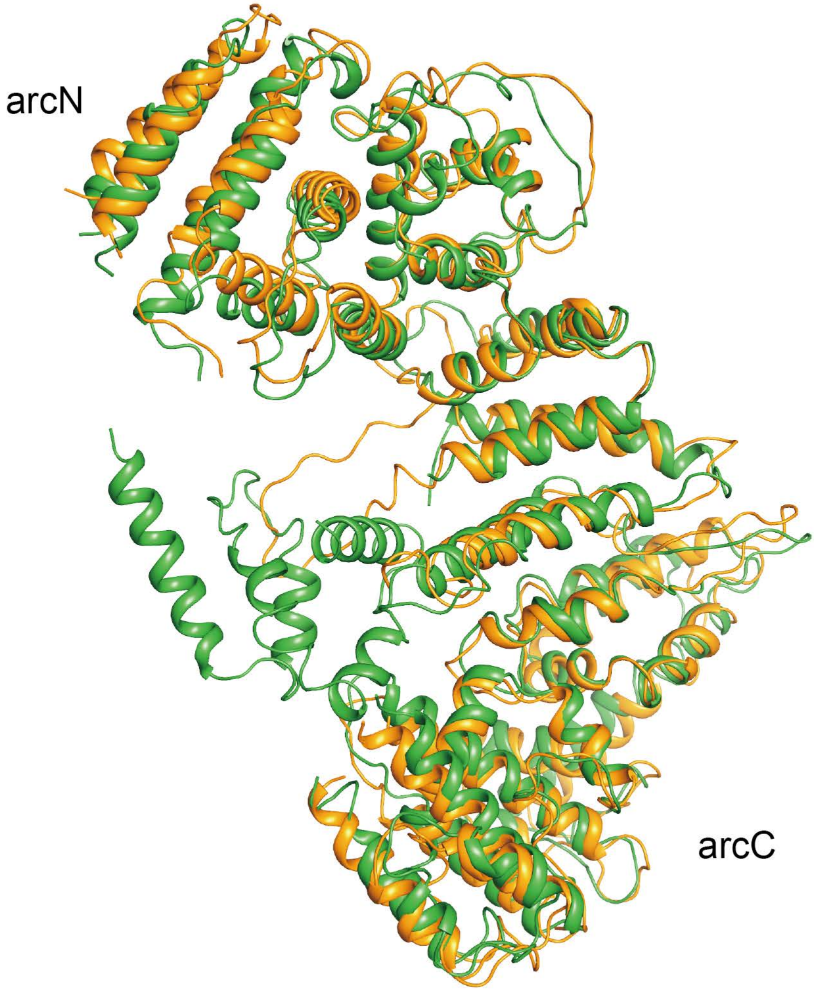


Supplementary Figure S5

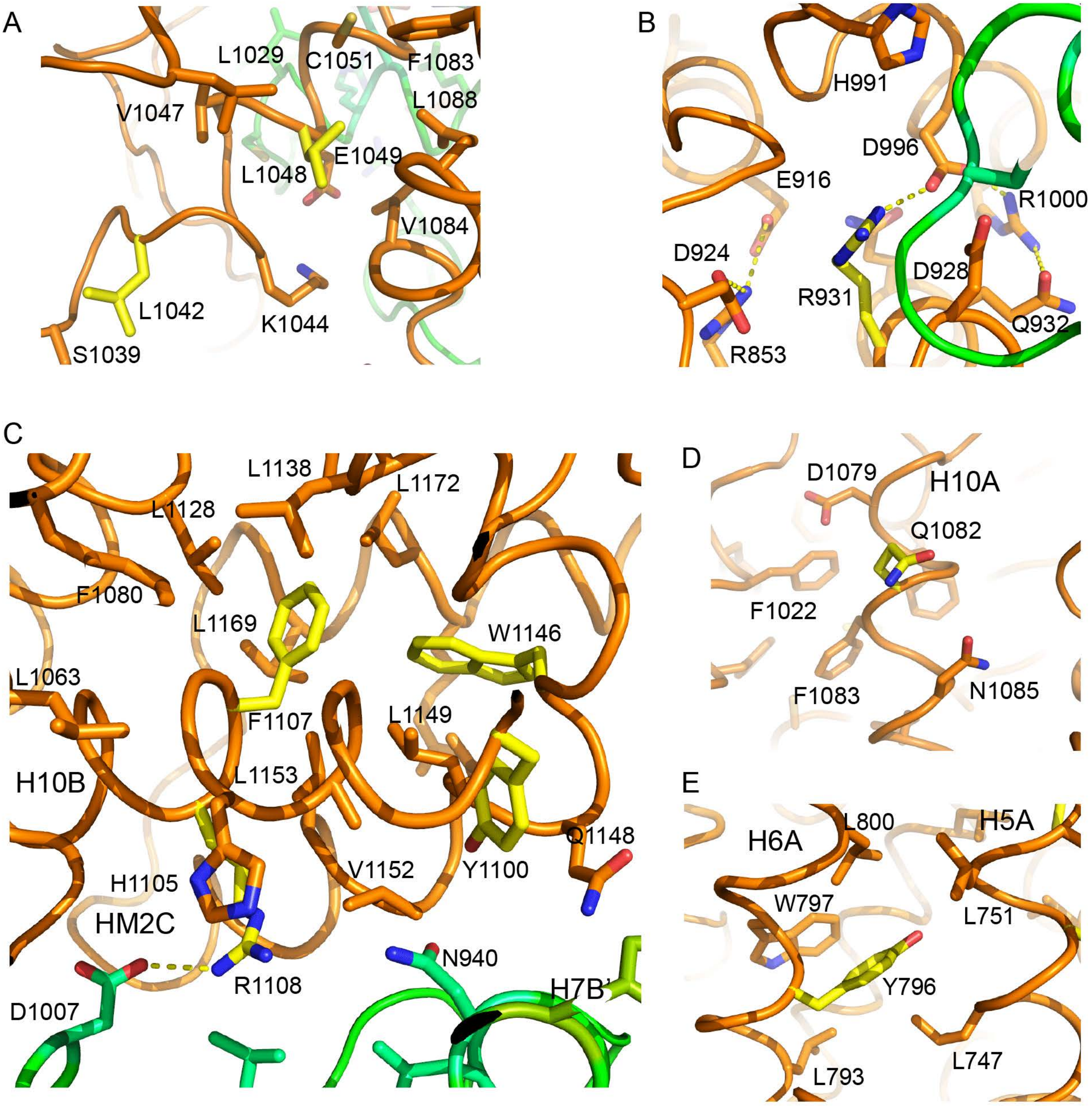


Supplementary Figure S6

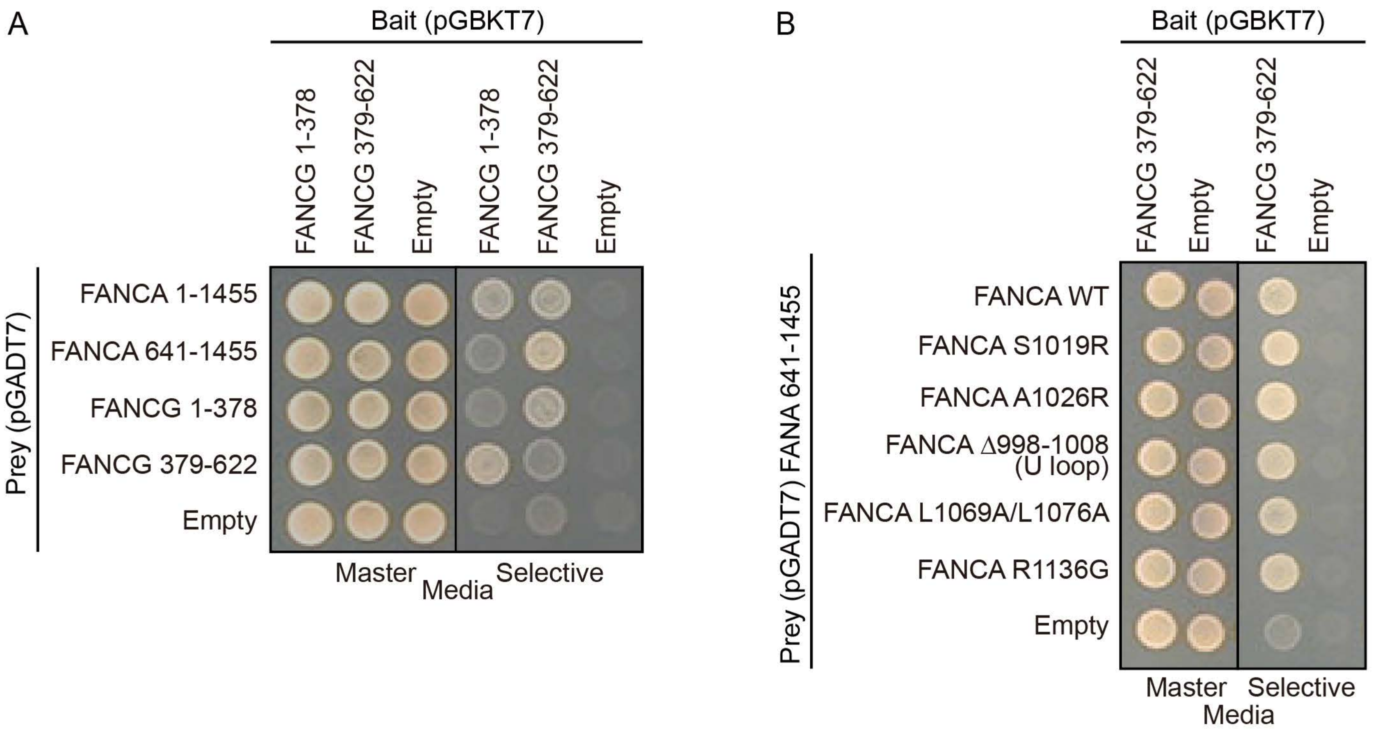


Supplementary Figure S7

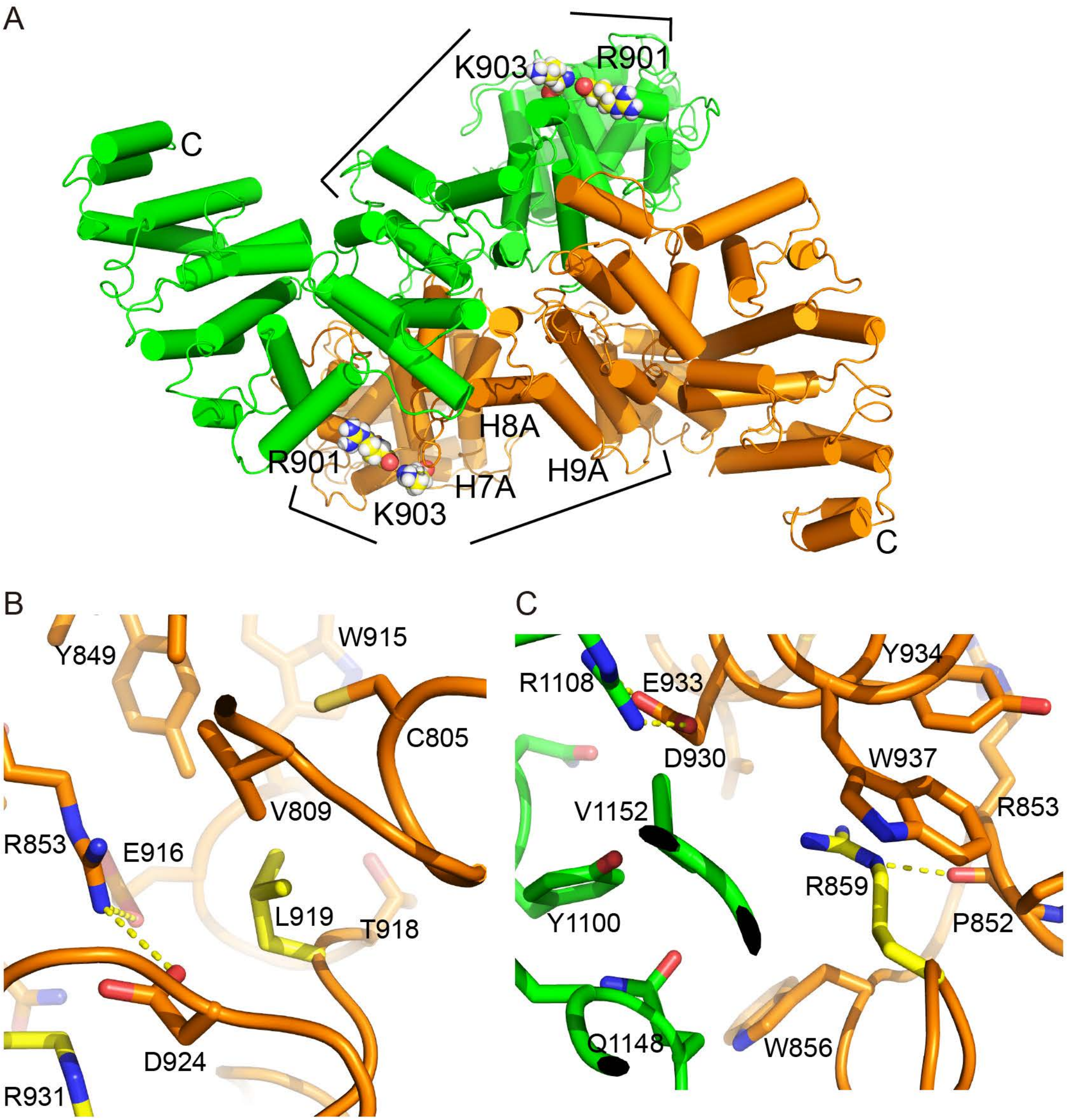


Supplementary Figure S8

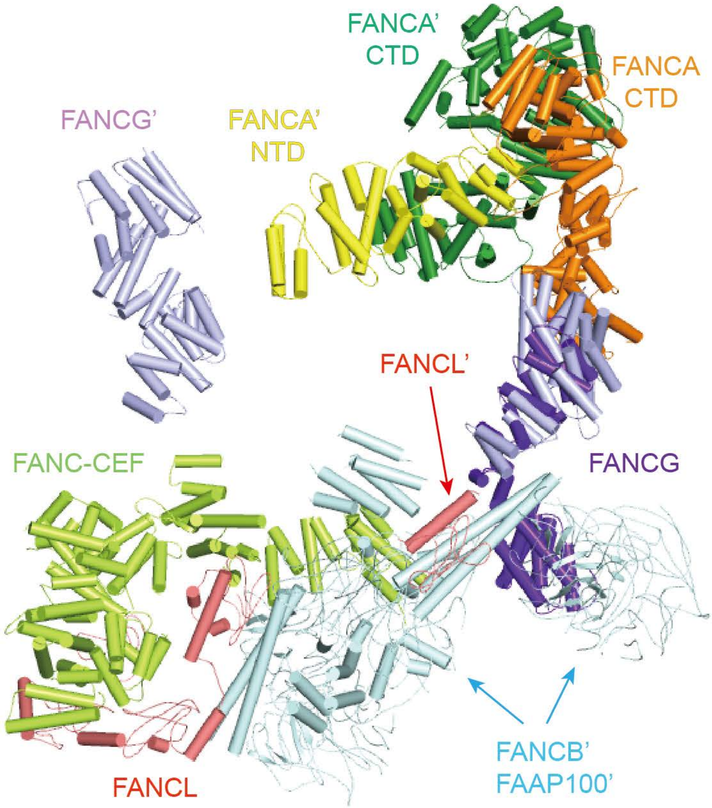


Supplementary Figure S9
