# Supplementary material for: Structural basis of the fanconi anemia-associated mutations within the FANCA and FANCG complex
Source: Nucleic Acids Res. 2020 Jan 31;48(6):3328–42. doi: 10.1093/nar/gkaa062 (PMC7102982; doi:10.1093/nar/gkaa062)
Supplement: gkaa062_Supplemental_Files [file gkaa062_supplemental_files.zip › Supplementary Tables S1-S3.docx]

Supplementary Table S1. Cryo-EM data collection, refinement and validation statistics

|  | FANCA CTD  residues  631-1402 | ^a^ FANCA CTD  residues  530-1402 | ^b^ FANCA-  FANG Complex | ^c^FANCA’-FANCG’ complex |
| --- | --- | --- | --- | --- |
| **Data collection and processing** |  |  |  |  |
| Magnification | 47,000 |  |  |  |
| Voltage (kV) | 300 |  |  |  |
| Total electron exposure (e-/ Å^2^) | 30 |  |  |  |
| Defocus rage (μm) | −1.5 to −2.5 |  |  |  |
| Pixel size (Å) | 1.4 |  |  |  |
| Symmetry imposed | C1 | C1 | C1 | C1 |
| Initial particle images (no.) | 1,523,728 | 1,523,728 | 1,523,728 | 1,523,728 |
| Final particle images (no.) | 306,460 | 150,141 | 89,678 | 64,905 |
| Map resolution (Å) | 3.35 | 3.46 | 4.59 | 4.84 |
| FSC threshold | 0.143 | 0.143 | 0.143 | 0.143 |
| **Refinement** |  |  |  |  |
| Model resolution (Å) | 3.8 | 4.0 |  |  |
| FSC threshold | 0.5 | 0.5 |  |  |
| Model resolution range (Å) |  |  |  |  |
| Map sharpening B-factor (Å) | -100.6 | -148.6 |  |  |
| Model composition |  |  |  |  |
| Nonhydrogen atoms | 11,424 | 11,688‬ |  |  |
| Protein residues | 1,456 | 1518 |  |  |
| Ligand | 0 | 0 |  |  |
| B factors (Å^2^) |  |  |  |  |
| Protein | 30.00 | 29.80 |  |  |
| Ligand | 0 | 0 |  |  |
| R.m.s. deviations |  |  |  |  |
| Bond lengths (Å) | 0.005 | 0.005 |  |  |
| Bond angles (°) | 0.616 | 0.775 |  |  |
| **Validation** |  |  |  |  |
| MolProbity score | 1.83 | 1.83 |  |  |
| Clashscore | 3.68 | 4.07 |  |  |
| Poor rotamers (%) | 0 | 0 |  |  |
| Ramachandran plot |  |  |  |  |
| Favored (%) | 84.47 | 86.36 |  |  |
| Allowed (%) | 15.53 | 13.64 |  |  |
| Disallowed (%) | 0 | 0 |  |  |

^a-c^ Same data set was used as that of FANCA CTD (631-1402).

^b^ FANCA-FANCG Complex represents the complex between FANCA CTD and FANCG

^c^ FANCA’-FANCG’ complex represents the complex between FANCA’ NTD and FANCG’

Supplementary Table S2. Localization analysis of FANCA mutants by immunofluorescence

| Mutant | ^a^ Homogeneity (%) | ^b^ Nucleus (%) | ^c^ Cytoplasm (%) |
| --- | --- | --- | --- |
| WT | 7 (±0.01) | 91 (±0.43) | 2 (±0.41) |
| WT+MMC | 4 (±0.25) | 96 (±0.25) | 0 (±0) |
| L1069A/L1076A | 17 (±0.41) | 83 (±0.41) | 0 (±0) |
| L1069A/L1076A+MMC | 12.8 (±1.7) | 86.9 (±2.2) | 0.3 (±0.5) |
| 1-1373 | 0 (±0) | 0 (±0) | 100 (±0) |
| 1-1373+ MMC | 0 (±0) | 0 (±0) | 100 (±0) |
| 1-1409 | 0 (±0) | 0 (±0) | 100 (±0) |
| 1-1409+MMC | 0 (±0) | 0 (±0) | 100 (±0) |
| R1055W | 0 (±0) | 0 (±0) | 100 (±0) |
| R1055W+MMC | 0 (±0) | 0 (±0) | 100 (±0) |
| W1302R | 5 (±2.1) | 0 (±0) | 95 (±2.1) |
| W1302R+MMC | 1 (±0.1) | 0 (±0) | 99 (±0.1) |
| L1082P | 1 (±0.1) | 0 (±0) | 99 (±0.1) |
| L1082P + MMC | 0 (±0) | 0 (±0) | 100 (±0) |
| F1262L | 44 (±1.1) | 0 (±0) | 56 (±1.1) |
| F1262L+ MMC | 32 (±0.6) | 0 (±0) | 68 (±0.6) |
| M1360I | 0 (±0) | 0 (±0) | 100 (±0) |
| M1360I + MMC | 0 (±0) | 0 (±0) | 100 (±0) |

^a^ Homogeneity, cytoplasm=nucleus; ^b^ Nucleus, nucleus dominant; ^c^ Cytoplasm, cytoplasm dominant. Standard error of the mean values from the two independent experiments are shown in the parenthesis.

Supplementary Table S3. FANCA CTD modelled as poly-alanine chains or disordered regions

| FANCA CTD modelled by the map at 3.35 Å | | | |
| --- | --- | --- | --- |
| Residues | Wild type sequence | Modelled sequence | |
|  |  | FANCA CTD | FANCA’ CTD |
| 631 | E | Wild type sequence | Not modelled |
| 666-669 | KLIA | Poly-alanine | Wild type sequence |
| 670-692 | VMGEQKVDDDQVAAKFLKLEDGA | Not modelled | Not modelled |
| 693-697 | QLDIQ | Poly-alanine | AADIQ |
| 973-988 | RHIQHSEWESRNISML | Wild type sequence | Not modelled |
| 1342 | V | Wild type sequence | Poly-alanine |
| 1343-1354 | DGSPLSSLSAKS | Not modelled | Not modelled |
| 1355 | H | Poly-alanine | Not modelled |
| 1356-1360 | HLDSH | Poly-alanine | Poly-alanine |
| 1361 | D | Wild type sequence | Poly-alanine |
| 1371-1402 | LCCVPKCQKPNSAILKKMLESWEEHDPELAAV | Poly-alanine | Poly-alanine |
| FANCA CTD modelled by the map at 3.46 Å | | | |
| Residues | Wild type sequence | Modelled sequence | |
|  |  | FANCA CTD | FANCA’ CTD |
| 530-614 | SQAHQDVEKALNIFENTGKIPASVMEASIFRRPYFTSRFLPALLTPRVLPAAPDALMLLIDSMKRADKIPTNMFNAYIEACEQEK | Not modelled | Wild type sequence |
| 615-619 | LRKQK | Not modelled | Poly-alanine |
| 620-630 | GRQQMDQSLPD | Not modelled | Not modelled |
| 631 | E | Poly-alanine | Not modelled |
| 632-669 | PLGILQSALSDLRPLVTDANKYEDVSAQVAVISEKLIA | Poly-alanine | Poly-alanine |
| 670-694 | VMGEQKVDDDQVAAKFLKLEDGAQL | Not modelled | Not modelled |
| 695-696 | DI | Not modelled | Wild type sequence |
| 863-868 | IRDDEE | Not modelled | Wild type sequence |
| 973-978 | RHIQHS | Wild type sequence | Not modelled |
| 979-987 | EWESRNISM | Not modelled | Not modelled |
| 988 | L | Wild type sequence | Not modelled |
| 1034-1040 | GKKELSN | Not modelled | Wild type sequence |
| 1341 | F | Poly-alanine | Wild type sequence |
| 1342 | V | Poly-alanine | Poly-alanine |
| 1343-1354 | DGSPLSSLSAKS | Not modelled | Not modelled |
| 1355 | H | Poly-alanine | Not modelled |
| 1356-1361 | HLDSHD | Poly-alanine | Poly-alanine |
| 1362-1370 | VFTCGRQFL | Poly-alanine | Wild type sequence |
| 1371-1402 | LCCVPKCQKPNSAILKKMLESWEEHDPELAAV | Poly-alanine | Poly-alanine |
